# Understanding Oxygen-Induced Reactions and Their Impact on n-Type Polymeric Mixed Conductor-Based Devices

**DOI:** 10.1021/acscentsci.4c00654

**Published:** 2024-11-19

**Authors:** Prem D. Nayak, Büsra Dereli, David Ohayon, Shofarul Wustoni, Tania Cecilia Hidalgo Castillo, Victor Druet, Yazhou Wang, Adel Hama, Craig Combe, Sophie Griggs, Maryam Alsufyani, Rajendar Sheelamanthula, Iain McCulloch, Luigi Cavallo, Sahika Inal

**Affiliations:** † Organic Bioelectronics Laboratory, Biological and Environmental Science and Engineering Division, King Abdullah University of Science and Technology (KAUST), Thuwal 23955-6900, Saudi Arabia; ‡ Physical Sciences and Engineering Division, KAUST Catalysis Center, KAUST, Thuwal 23955-6900, Saudi Arabia; § KAUST Solar Center, Physical Sciences and Engineering Division, KAUST, Thuwal 23955-6900, Saudi Arabia; ∥ Department of Chemistry, Chemistry Research Laboratory, University of Oxford, Oxford OX1 3TA, U.K.

## Abstract

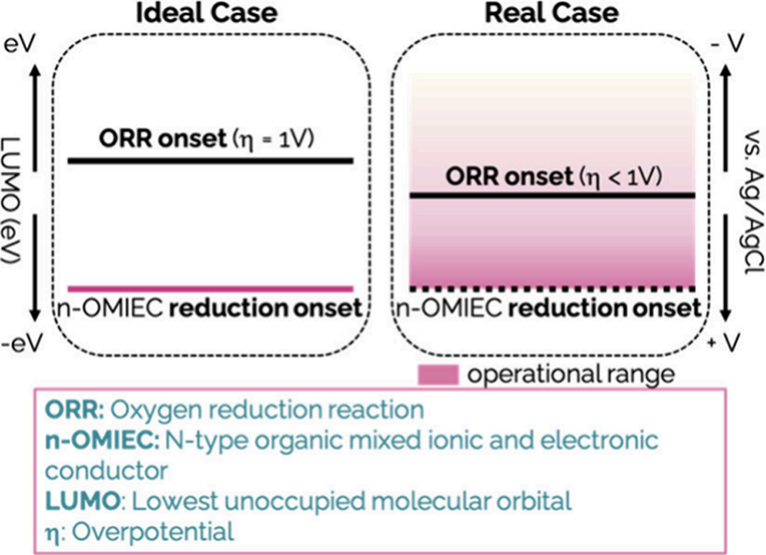

Electron transporting (n-type) polymeric mixed conductors are an exciting class of materials for devices with aqueous electrolyte interfaces, such as bioelectronic sensors, actuators, and soft charge storage systems. However, their charge transport performance falls short of their p-type counterparts, primarily due to electrochemical side reactions such as the oxygen reduction reaction (ORR). To mitigate ORR, a common strategy in n-type organic semiconductor design focuses on lowering the lowest unoccupied molecular orbital (LUMO) level. Despite empirical observations suggesting a correlation between deep LUMO levels, low ORR, and enhanced electrochemical cycling stability in water, this relationship lacks robust evidence. In this work, we delve into the electrochemical reactions of n-type polymeric mixed conductors with varying LUMO levels and assess the impact of ORR on charge storage performance and organic electrochemical transistor (OECT) operation. Our results reveal a limited correlation between LUMO levels and ORR currents, as well as the electrochemical operational stability of the films. While ORR currents minimally contribute to OECT channel currents under fixed biasing conditions, n-type films self-discharge rapidly at floating potentials in a capacitor-like configuration. The density functional theory analysis, complemented by X-ray photoelectron spectroscopy, underscores the critical role of backbone chemistry in controlling O_2_-related degradation pathways and device performance losses. These findings highlight the persistent challenge posed by ORR in n-type semiconductor design and advocate for shifting the focus toward exploring chemical moieties with limited O_2_ interactions to enhance operational stability and performance at n-type film/water interfaces.

## Abstract 2

The onset of oxygen reduction reaction for n-type organic mixed ionic-electronic conductors closely aligns with their reduction onset and can hardly be mitigated by frontier energy level adjustment.

## Introduction

Organic mixed charge conductor (OMIEC) films can simultaneously transport and couple ionic and electronic charges in their bulk.^1^ These soft solids undergo reversible reduction and oxidation reactions within the water-stable electrochemical window, making them attractive for applications such as bioelectronic actuators, sensors, green charge storage, and energy conversion devices. A key electronic device that capitalizes on the ionic charging phenomenon in OMIECs is the organic electrochemical transistor (OECT).^2,3^ In OECTs, an OMIEC film serves as the channel material, often paired with an aqueous electrolyte as the dielectric medium. Most OMIECs in OECTs are based on π-conjugated backbones with tethered oligo(ethylene glycol) (OEG) side chains utilized for ion solvation and transport.^4^ Hole-conducting (p-type) OMIECs have, so far, dominated OECT applications, surpassing the performance of their electron-transporting (n-type) counterparts.^5^ One factor contributing to the subpar performance of n-type semiconductors is the instability of electronic charge in ambient conditions.^6^ The redox potential of oxygen (O_2_) is often close to the lowest unoccupied molecular orbital (LUMO) of n-type polymers, acting as a trap or scavenger of polaronic states.^7,8^ The net result is a reduction in the number of mobile electrons available, which would otherwise contribute to the current output. Ensuring the stability of n-type organic field effect transistor (OFET) devices in air or water interfaces requires mitigating channel oxidation by O_2_ and H_2_O. Consequently, it has been proposed that n-type materials with a LUMO level deeper than ca. 4.0 eV (with respect to vacuum) are necessary for stable OFET operation. This guideline has persisted as a key principle for the design of n-type OMIECs, despite these materials and their devices (such as the OECT) working in markedly different environments (e.g., in air and inside an aqueous electrolyte) compared to OFETs.^5,9^

Recent research has suggested that deep LUMO levels may be advantageous for high OECT performance and stability. For example, the fluorination of thiophene units of a donor–acceptor type OMIEC lowered the LUMO level, resulting in n-type OECTs with enhanced operational stability compared to devices using an analog polymer with a shallower LUMO.^10^ This stability improvement was attributed to the incorporation of hydrophobic fluorine atoms, which curtailed undesired side reactions with O_2_. However, this modification also influenced the film’s hydration during doping and dedoping cycles, preventing the morphological changes typically induced by such cycling. Since both the film microstructure and the LUMO level are altered by fluorination, establishing a direct correlation between device stability and performance enhancement through LUMO adjustment in this specific case is not straightforward. Another study compared the OECT performance of n-type polymers with distinct LUMO levels achieved by extending the backbone coplanarity. However, this work did not yield conclusive evidence for a direct connection between improved stability and reduced parasitic reactions stemming from deeper LUMOs.^11^

Moreover, the notion that deep(er) LUMO levels guarantee ambient stability may not be entirely accurate, even for OFETs.^12,13^ Di Pietro et al. highlighted the importance of considering the energetics of specific chemical sites within polymer structures and examining their interactions with O_2_ and H_2_O_2_, rather than focusing solely on LUMO.^14^ They critiqued the conventional approach of simplifying polymer air stability to a single value representing the polaron energy on the polymer chain, with its onset defined by the LUMO. Specifically, they found that P(NDI2OD-T2), a copolymer comprising a bithiophene (T2) donor and a naphthalene diimide (NDI) acceptor unit with alkyl side chains, experienced degradation in air due to a stabilizing reaction (forming a lower energy product) with O_2_ and water. This reaction was driven by the presence of a fused benzene ring structure rather than being contingent on LUMO energy levels. Furthermore, while the importance of molecular design—such as specific functional groups, stereochemistry, and the introduction of steric hindrances—on a material’s chemical reactivity has been well-established in the fields of organocatalysis^15−17^ and intercalation-based energy storage devices,^18,19^ these concepts have not been explored in detail for organic mixed conductors, which are susceptible to various faradaic reactions.

Another important distinction of n-type OMIECs is that they usually operate in aqueous media rich with H^+^ ions, significantly altering how O_2_ interacts and reacts.^20^ Recent studies found that ORR occurs in n-type OMIECs with LUMO in the range of 4.1 to 4.3 eV. One of these examined the ORR capability of the n-type poly(benzimidazobenzophenanthroline) (BBL), revealing a two-step electron tunneling process during ORR, which leads to H_2_O generation with an intermediate step involving H_2_O_2_ production.^21^ Another study focusing on a glycolated NDI-T2-based copolymer film found that ORR occurred through an outer-sphere electron transfer process to form H_2_O_2_.^22^ Considering these findings, for n-type OECT operation in water, ORR seems unavoidable due to the overlapping conductivity modulation and ORR electrochemical window in n-type OMIECs with LUMOs typically around 3.8 eV-4.3 eV.^23^ It is thus crucial to understand whether ORR affects device performance. Furthermore, when discussing ORR, degradation is frequently mentioned in the same breath. Nevertheless, instances exist where ORR is present, yet no degradation occurs.^21,22^ Consequently, studies that aim to distinguish between these phenomena and understand why devices can be stable despite ORR are warranted.

In this work, we investigated the interactions between O_2_ and three ambient-stable n-type OMIECs widely used in OECTs. Using linear voltammetry conducted via a rotating disc electrode and Tafel slope analysis, we quantified ORR contributions to the reduction currents collected from the films. Our findings revealed that while all films exhibited ORR, variations in ORR currents did not correlate with the LUMO levels of the polymers. Electrochemical doping levels of the films remained unaffected by ORR, as validated through UV–vis spectroscopy studies. OECT performance evaluation indicated that ORR primarily impacted the gate currents without reducing the channel currents. Further investigation into the ORR products revealed that the polymers generated a mixture of H_2_O and H_2_O_2_, and ORR during device operation changed the pH of the environment surrounding the device. Additionally, we foud that ORR adversely affected capacitor-like charged films, causing them to self-discharge instantly upon removal of the applied potential. Even n-type polymers with much deeper LUMO levels underwent ORR and some even degraded due to interactions with O_2_. Through density functional theory (DFT) calculations and X-ray photoelectron spectroscopy (XPS), we determined that O_2_-polymer interactions are predominantly influenced by specific chemical moieties in the polymer’s molecular structure, and the nature of these interactions determines the air stability of the film. Our findings lead to two important conclusions: 1) LUMO adjustments are unlikely to eliminate ORR of n-type OMIECs operating in ambient aqueous media, and 2) depending on the polymer chemical structure, interactions between n-type OMIECs and O_2_ can lead to film degradation or the formation of ORR products that may impede device functionality, while other interactions may be benign, not affecting electrochemical cycling stability.

## Results

### ORR Evaluation Using a Rotating Disc Electrode (RDE)

**Figure 1:**
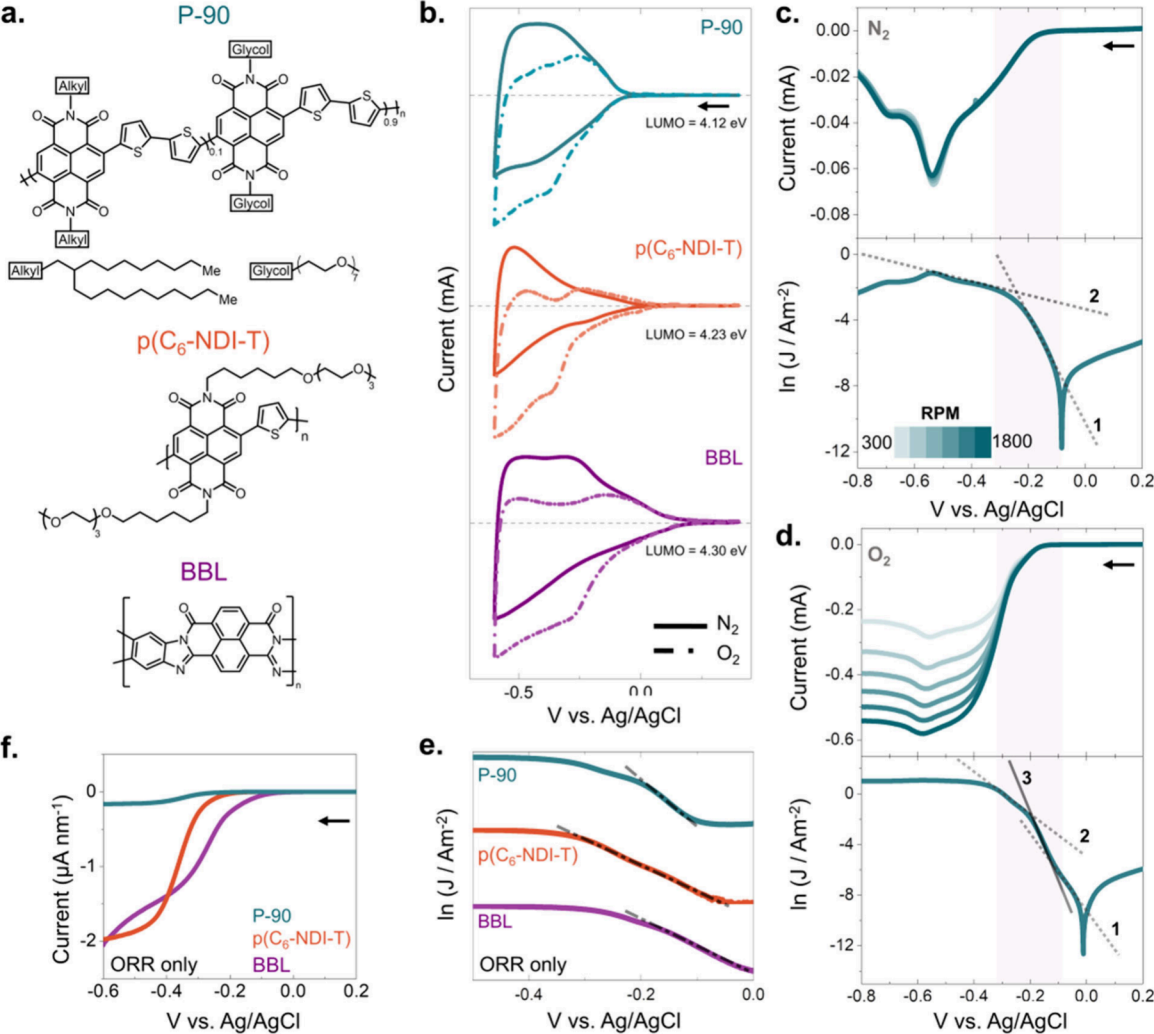
(a) Chemical structures of n-type OMIECs investigated in this study: P-90, p(C_6_-NDI-T), and BBL. (b) Cyclic voltammetry curves of the films coated on a glassy carbon electrode recorded at a scan speed of 50 mV/s in N_2_-saturated and O_2_-saturated PBS. The arrow indicates the scan direction. (c) LSV curves of a P-90 film recorded in (c) N_2_-saturated PBS and (d) O_2_-saturated PBS at different rotation speeds (from 300 to 1800 rpm). The shaded area in shows the potential range used in the Tafel analysis. Tafel plots (natural log of current of the LSV curve recorded at 1200 rpm) are shown at the bottom panel. Different slopes in the relevant regions are shown with numbered lines. (e) Tafel plot obtained by subtracting the N_2_-saturated plot from the O_2_-saturated one. (f) Thickness normalized ORR currents. All scans were performed in PBS (ca. 0.15 M) at a 5 mV/s scan rate to ensure steady-state current collection. The arrows indicate the voltage scan direction.

The chemical structures of the three polymers we chose to investigate in this study are shown in Figure 1a. P-90 is an n-type OMIEC with a naphthalene tetracarboxylic diimide and bithiophene (NDI-T2) based backbone. The NDI unit is functionalized with a branched alkyl or linear OEG side chain, and P-90 is a random copolymer that bears 10% of the alkylated NDI-T2 monomer.^4^ The p(C_6_-NDI-T) also has an NDI backbone, coupled instead with a single thiophene as the donor unit, and is entirely composed of OEG side chains.^24,25^ BBL is touted among the best-performing n-type OMIECs devoid of EG or any other side chains in the structure.^26,27^ Studying p(C_6_-NDI-T), which has a chemical structure similar to P-90, and BBL with a different structure than P-90 and BBL will allow us to determine if O_2_ interactions recorded with one polymer are extrapolatable to other n-type OMIECs and to understand which chemical motif (if at all) dictates reactions with O_2_. All three polymers have similar LUMO values, ranging from 4.12, 4.23, and 4.30 eV, for P-90, p(C_6_-NDI-T), and BBL, respectively (Table S1).

All polymer films generate currents when biased in phosphate-buffered saline (PBS) at negative voltages vs Ag/AgCl, with clear reduction and oxidation peaks (Figure 1b). The reduction and oxidation currents suggest that the films couple electrons with cations, i.e., n-doped, and they can be dedoped as the cations are ejected back to the electrolyte. While the first cyclic voltammetry (CV) curve differs from the subsequent ones, we note the stability of the materials against successive doping and dedoping cycles (Figure S1). Importantly, the CV curves collected without O_2_ in PBS differ from those collected when O_2_ is present. To differentiate the impact of the possible electrochemical reactions of the films with O_2_ on the reduction currents collected, we used the RDE technique. RDE is often used to mimic the electrochemical properties of microelectrodes due to their ability to reach a mass transfer-controlled steady state quickly.^31−33^ During the RDE experiment, we conducted linear sweep voltammetry (LSV) and scanned the films at various rotation speeds with voltages applied in the reduction regime. We collected the current in PBS saturated with O_2_ and PBS free of O_2_. Figure 1c and **d** shows the LSV curves during a reduction sweep for P-90 (from 0.2 V to −0.8 V vs Ag/AgCl) in N_2_ and O_2_ saturated PBS, respectively. LSV shapes do not change with rotation speed in N_2_ saturated electrolyte, indicating the absence of any mass transfer-dependent faradaic reaction (e.g., hydrogen evolution reaction (HER) or ORR). However, when O_2_ is present, the reduction currents reach higher values than in N_2_-saturated conditions and increase as the disk rotates faster. The increase in current values and its dependence on rotation speeds evidence ORR and its contribution to the overall current that we recorded in the ambient environment. LSV curves obtained at different rotational speeds overlap in the kinetic-controlled regime (i.e., mass transfer is not limiting), reaching the mixed regime (kinetics and mass transfer dependent) and, finally, a plateau at relatively high negative voltages dependent on rotational speeds (Figure 1d). The bottom panels in Figure 1c and d show the Tafel plot representation of the data just above them, i.e., the logarithm of the current density versus the overpotential. This relationship is valid in regions where mass transport is not limited.^28^ Focusing on this regime in N_2_ saturated conditions, we observe two linear regions. We attribute the first steep slope at low potentials to the charging of the polymer surface readily exposed to the electrolyte cations and the second slope at higher potentials to the doping of the bulk film. In the presence of O_2_, the second region of the LSV is now composed of two linear portions, which could be due to the ORR occurring in the same potential range as the doping. LSVs and subsequent Tafel slope analysis of p(C_6_-NDI-T) and BBL in N_2_- and O_2_- saturated electrolytes are shown in Figure S2 and exhibit similar behavior.

To quantify the ORR current, we subtracted the current measured in the N_2_-saturated electrolyte from the current recorded in the O_2_-saturated electrolyte. After converting the resulting current to a logarithmic scale, we calculated the Tafel slope of the isolated ORR current. Interestingly, only one Tafel slope is observed (Figure 1e), indicating that the ORR and electrochemical doping are independent processes and that ORR occurs parallel to electrochemical doping without affecting doping dynamics. We also observe that the optical features of the film addressed at a particular doping potential are identical in O_2_-bearing or O_2_-free electrolytes, indicating that ORR does not affect the electrochemical state that the film reaches (Figure S3). Finally, Figure 1f shows the thickness-normalized ORR-only currents for the three polymers, revealing that BBL, despite its slightly deeper LUMO, generates the largest ORR current.

### The Impact of ORR on OECT Steady-State Performance

**Figure 2:**
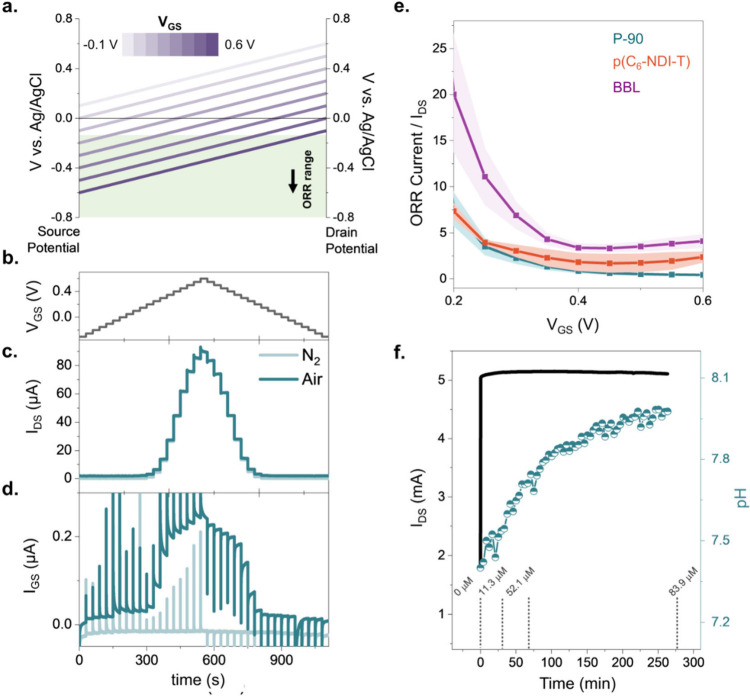
(a) Electrochemical potential distribution across the channel during the application of V_GS_ increasing from −0.1 to 0.6 V at V_DS_ = 0.5 V. The highlighted region corresponds to the ORR window. We assume a linear potential distribution from the source to the drain and that none of the applied V_GS_ drops at the gate–electrolyte interface. (b) V_GS_ vs time profile and the resulting (c) I_DS_ and (d) I_GS_ of P-90 OECTs recorded in ambient and O_2_-free PBS. V_DS_ = 0.5 V. (e) ORR currents vs V_GS_. The error values were derived from at least 3 channels. (f) Real-time monitoring of pH and H_2_O_2_ concentrations in the electrolyte during the device operation. V_GS_ = 0.5 V and V_DS_ = 0.5 V were applied for ca. 300 min. The OECT channel current was recorded simultaneously.

Having confirmed that all three materials undergo ORR and that ORR does not hinder electrochemical doping in any of the three polymers, we proceeded to investigate how O_2_ influences the operation of OECTs incorporating these films in the channel. Figure 2a illustrates the potential changes across the channel as we scan the gate-source voltage (V_GS_) from −0.1 to 0.6 V while keeping the drain-source voltage (V_DS_) constant at 0.5 V. As V_GS_ increases, a larger fraction of the channel enters the ORR regime. We started the measurements at ∼ −0.1 V as an estimate for ORR threshold considering the ORR onsets of P-90, p(C_6_-NDI-T), and BBL, which are −0.16 V, −0.22 V, and −0.13 V vs Ag/AgCl, respectively.

P-90 source-drain current (I_DS_) in N_2_- and air-saturated electrolytes are practically identical at all voltages, as shown in Figure 2c. ORR-current is compensated by the gate electrode, evidenced by the higher gate currents (I_GS_) of the same device operating in air (Figure 2d). The other two polymers also have channel currents independent of O_2_ and gate currents larger in air (Figure S4). Note that for BBL, this statement is valid only until V_GS_ = 0.45 V, beyond which the film shows some atypical behavior in N_2_-saturated conditions as the currents flatten due to HER (Figure S4c, see also Figure S2c for HER currents negative of −0.4 V vs Ag/AgCl). These results align with our RDE findings, which suggested that ORR and electrochemical doping are two independent events, provided that electrochemical potentials are maintained. We extracted the ORR currents (*I*_*GS,O*_2__-*I*_*GS,N*_2__) (normalized by the corresponding I_DS_ to eliminate the effect of channel conductivity) as reported in Figure 2 e, showing that the BBL OECT generates largest ORR currents compared to P-90 and p(C_6_-NDI-T) devices, in agreement with RDE results (Figure 1 d).

### Analysis of ORR Products

ORR can occur via two main pathways, producing hydrogen peroxide (O_2_ + 2H^+^ + 2*e*^–^ → H_2_O_2_, two-electron process) or H_2_O (O_2_ + 4H^+^ + 4*e*^–^ → 2H_2_O, four-electron process), or both at the same time via a sequential pathway.^29,30^ It is important to analyze reaction products as they can impede charge retention and/or lower device stability by interacting with the device components.^31^ Moreover, H_2_O_2_ accumulation in biological environments can be deleterious. We tested the three polymers using a rotating ring disc electrode (RRDE) to determine the composition of ORR products at various potentials and pH conditions. We found that all our films produce H_2_O_2_ and H_2_O in mixed quantities (Figure S5). P-90, for example, preferentially undergoes the four-electron process at natural and low pH, while BBL and p(C_6_NDI-T) are prone to the two-electron process. We found a pH dependence of the reaction product nature and quantity, with more H_2_O production when the pH decreases (see Section 1 of the Supporting Information for a discussion on ORR reaction products). Notably, since ORR consumes H^+^ ions regardless of the pathway taken, continuous operation of n-type OECTs is expected to increase the pH of the measurement solution. In fact, for a P-90 OECT, after 300 min of continuous operation at ORR enabling conditions (V_GS_ = V_DS_ = 0.5 V), pH increased from 7.4 to 8, with the film producing around 80 μM of H_2_O_2_ while working in 1 mL of electrolyte (Figure 2f).

### The Impact of ORR on Electrochemical Self-Discharging

**Figure 3:**
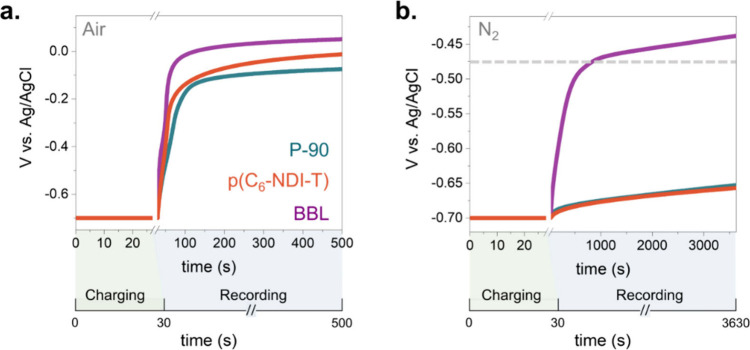
Change in the OCP of n-type OMIECs after they were held at a −0.7 V vs Ag/AgCl for 30 s in PBS under (a) air and (b) N_2_ conditions. The dotted line refers to the HER onset for BBL.

N-type OMIECs are increasingly being used to develop devices for charge storage^32^ and neuromorphic computing.^33^ The performance of devices in both applications depends on how well the polymer can retain its charged/doped state once the doping bias is removed. For a charge storage device, the material must not self-discharge through side reactions when no power is drawn. Similarly, for neuromorphic devices, nonvolatile memory behavior requires the doping state of the OMIEC to be retained once the voltage source is removed. So far, such device applications using n-type polymers have been demonstrated mostly inside a glovebox or with deoxygenated electrolytes,^33−35^ introducing operational and production-level challenges. To evaluate the effect of ORR on the discharging behavior of n-type OMIECs, we charged the films at −0.7 V vs Ag/AgCl for 30 s and monitored their respective open circuit potential (OCP) evolution. We used an RDE setup (2000 rpm) to ensure equivalent O_2_ availability across the experiments, while control experiments were performed in a deoxygenated electrolyte in an N_2_-filled glovebox (5 ppm of O_2_). Figure 3a shows that O_2_ quickly dedoped all three n-type OMIECs to their original OCP values. In the absence of O_2_, P-90 self-discharged by only 0.05 V even after 1 h, and p(C_6_-NDI-T) behaved exactly like P-90, suggesting the ORR-induced self-discharge in ambient conditions (Figure 3b). When the doped BBL was left to self-discharge in the N_2_-saturated electrolyte, the OCP decreased by 0.25 V in the first 500 s, after which it followed similar kinetics as of P-90 and p(C_6_-NDI-T). This faster self-discharge of BBL can be attributed to the HER until the electrode potential decreases below the HER onset (ca. −0.45 V vs Ag/AgCl (3 M NaCl)), after which its self-discharge slows down. Furthermore, when we varied the O_2_/N_2_ ratio in the environment, we observed that O_2_-driven self-discharge began at an O_2_ concentration of 4%, much lower than the 21% O_2_ present in air (Figure S6). Since O_2_ is more soluble in water than in N_2_,^36^ the concentration of O_2_ in ambient water is likely to be greater than 21%, allowing the O_2_-related effects to manifest at lower ambient O_2_ concentrations.

### DFT Calculations

We used DFT calculations to understand how our n-type OMIECs interact with O_2_ and correlate these interactions to the ORR currents generated by each material. We used a dimer model for each polymer and first computed the one-electron reduction potentials (Table 1). By comparing these values to the predicted reduction potential of ^3^O_2_ to the ^2^O_2_^•–^ radical anion, we aimed to see which species get reduced first. The DFT-predicted reduction potential for ^3^O_2_ to ^2^O_2_^•–^ (−0.53 V vs SHE) deviates from the experimental value (−0.33 V vs SHE)^37^ by 0.2 V, which is within the typical error of DFT calculations of this kind.^38^ From the computed reduction potentials, we found that the one-electron reduction potential of our polymers is lower than or close to the reduction overpotential of molecular O_2_, suggesting that the polymer is reduced first, followed by a subsequent electron transfer to molecular O_2_. When we conducted a similar analysis using a trimer model (Table S2, Figure S7), the predicted reduction potentials remained nearly the same compared to the dimer models. The largest difference was around 90 mV, observed between the p(C_6_-NDI-T) dimer (−0.54 V vs SHE) and the trimer models (−0.63 V vs SHE).

**Table 1:** Predicted One-Electron Reduction Potential of the Molecular O_2_ and the Three Dimer Models and the DFT-Calculated Binding Energy of Molecular O_2_ with the Reduced Dimers in Aqueous Solution^a^

reduction reaction	*E*^0^ vs SHE (V)	reduced film	O_2_ binding energy (eV)
^1^P-90 + e^–^ → ^2^P-90^•–^	–0.49	^2^P-90^•–^	0.63
^1^p(C_6_-NDI-T) + e^–^ → ^2^p(C_6_-NDI-T)^•–^	–0.54	^2^p(C_6_-NDI-T)^•–^	0.61
^1^BBL + e^–^ → ^2^BBL^•–^	–0.48	^2^BBL^•–^	1.56
^3^O_2_ + e^–^ → ^2^O_2_^•–^	–0.53		

a Calculated at the ωB97x-D/6-31+G(d)//ωB97x-D/6-311++G(d,p) level of theory.

Next, we investigated the binding mode of O_2_ to the reduced dimers and the subsequent electron transfer reactions once the film is charged. The reactions are outlined in eqs 1-6, with the chemical structures of the possible oxygenated species shown in Figure S8 for P-90. The computed free energy diagram indicates that O_2_ has similar binding energies with the reduced dimer models of P-90 and p(C_6_-NDI-T) (Table 1), likely due to their comparable backbone structures. The predicted energy for O_2_ binding to the thiophene adjacent to the end thiophene in the dimer model is 0.8 kcal/mol higher than the energy for O_2_ binding to the terminal thiophene in an end-on mode. Meanwhile, the energy for O_2_ binding to the thiophene in the middle of the dimer model is slightly higher, i.e., 6.9 kcal/mol more than for the end-on mode. The end-on mode binding of O_2_ at the terminal alkene site was predicted to be the most stable, whereas monodentate binding was significantly less stable by 22.8 kcal/mol. Binding to the NDI acceptor unit yielded the least stable isomer, with an energy difference of 27.1 kcal/mol, suggesting that the thiophene unit serves as the electron carrier to the O_2_ substrate. It is important to note that the terminal alkene positions (in both end-on and monodentate modes) do not reflect the real system and are artifacts of using dimer models. Therefore, O_2_ binding to the thiophene at the center of the dimer unit or adjacent to the terminal thiophene is energetically favorable and most likely the real scenario. Binding at the NDI unit is unfavorable and we hypothesize that it could result in chemical degradation via ring opening, which would be contradicting with the stable electrochemical performance of P-90 and p(C_6_-NDI-T) over multiple cycles (Figure S1). The electron transfer upon reduction is thus expected to originate from the polymer’s donor unit rather than the NDI.

For P-90, the formation of an oxygenated polymer complex, POO^•–^, through an electron transfer from the reduced dimer, P^•–^, to O_2_, is endergonic by 0.63 eV (**2**), and this overpotential can be overcome in ambient conditions. POO^•–^ can accept one electron and one proton simultaneously (considering neutral pH conditions) to form the POOH^–^ intermediate with 0.07 eV of work (**3**). Subsequent proton-coupled electron transfer (PCET) yields PO^•–^ at −3.08 eV with the release of one H_2_O molecule (**4**). Further, the PCET step leads to the formation of POH^–^ intermediate found at −3.92 eV (**5**). Successive protonation of POH^–^ to P regenerates the neutral OMIEC dimer, and two H_2_O molecules are released as a result of a 4e^–^/4H^+^ reaction mechanism **(6)**. These steps describe how P-90 loses its electrons to O_2,_ and we find the same pathway with slightly altered energies for p(C_6_-NDI-T). The constant gate-source biasing in an OECT compensates for these losses in current. However, when the polymer is in a charged capacitor-like condition, the ORR leads to a loss of cell potential.
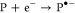
1
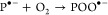
2
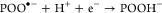
3
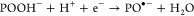
4
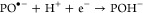
5
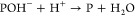
6

**Figure 4:**
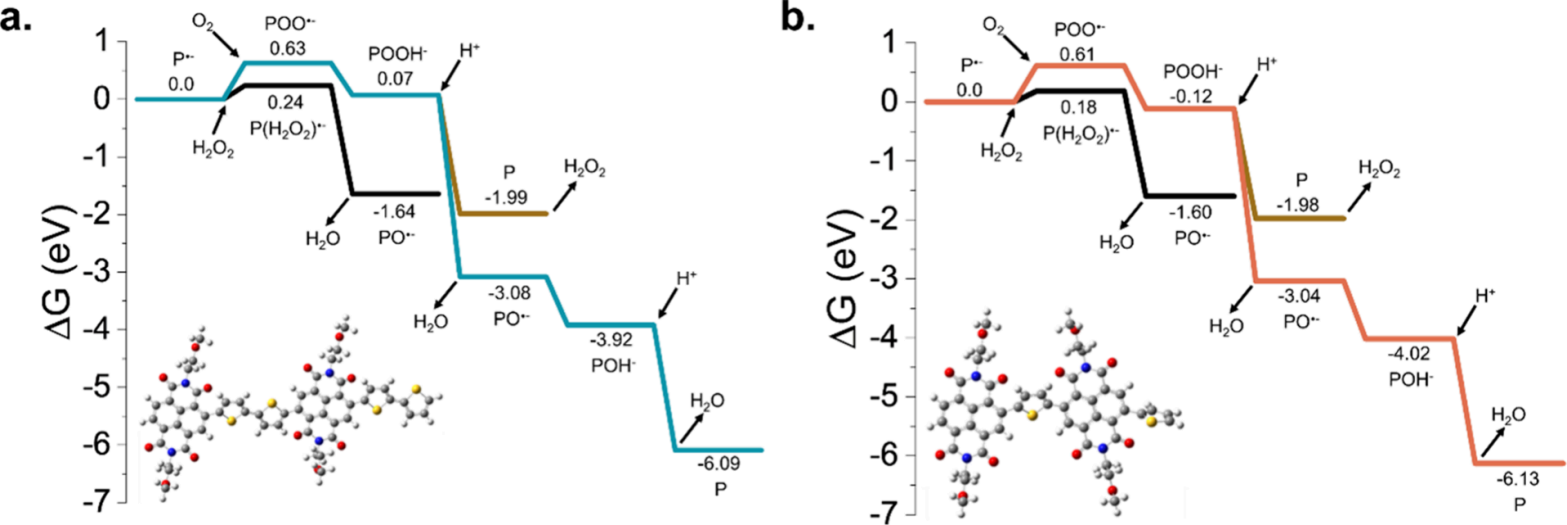
Free energy diagram vs reaction coordinate of the reduced dimer (P^•–^) models for (a) P-90 and (b) p(C_6_-NDI-T) for possible reaction pathways. The isolated molecular O_2_ and reduced dimer are taken as zero energy.

Under electrochemical operation (either under constant bias or capacitor-like charged state), P-90 and p(C_6_-NDI-T) react with molecular O_2_ and cause the reduction of O_2_ to H_2_O (O_2_ + 4H^+^ + 4*e*^–^ → 2H_2_O). The 2e^–^/2H^+^ reaction mechanism forming H_2_O_2_ (O_2_ + 2H^+^ + 2*e*^–^ → H_2_O_2_) is thermodynamically less favorable (Δ*G* = −1.44 eV vs Δ*G* = −5.54 eV for H_2_O formation) due to the energetic cost of successive protonation steps of POOH^–^, as shown in Figure 4a and 4b. Note that even though our proposed mechanism agrees with the experimental results for P-90, p(C_6_-NDI-T) was observed to produce an equal amount of H_2_O and H_2_O_2_.

According to DFT calculations, interactions with O_2_ are very different for BBL. Contrary to P-90 and p(C_6_-NDI-T), DFT calculations do not predict the stable formation of an O_2_:BBL intermediate complex (POO^•–^), as the predicted formation energy of 1.56 eV is not surmountable at ambient conditions (Table 1). The predicted free energy of binding to the NDI unit is 2.01 eV (46.5 kcal/mol), ruling out the BBL-O_2_ complexation on the NDI unit. We suggest that ORR in BBL involves the generation of free ^2^O_2_^•–^ radical anion through an outer-sphere mechanism, in agreement with another study.^21^ Moreover, we experimentally validated the O_2_-induced reaction paths predicted by DFT by performing sequential step-chronoamperometry measurements in dry conditions (no PBS, hence no H^+^ ions) in the absence and presence of O_2_ (see Section 2 of the Supporting Information and Figures S9–10).

### The Effect of ORR on the Stability of n-Type OECTs in Air and the Origins of Degradation

**Figure 5:**
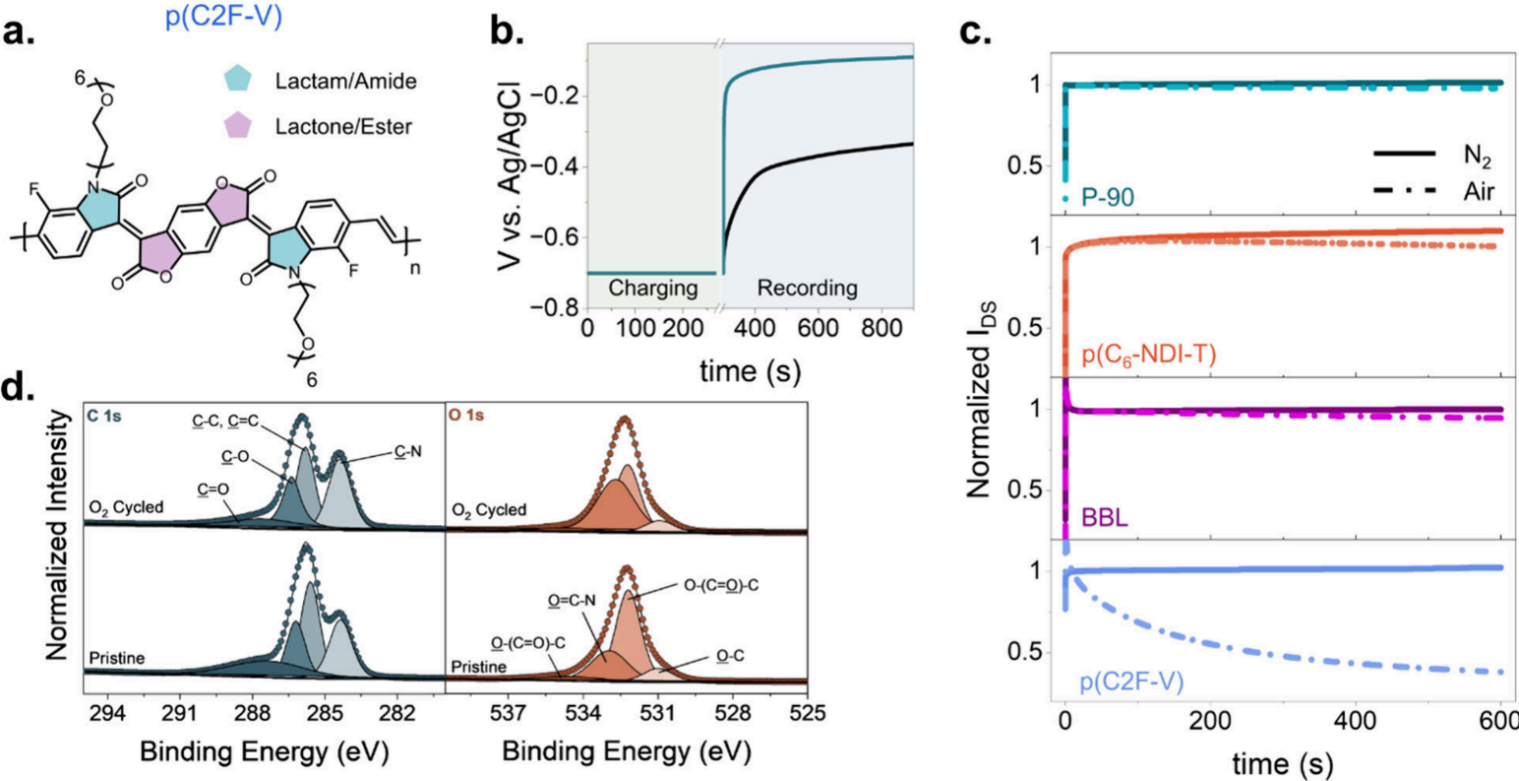
(a) The chemical structure of the deep LUMO polymer p(C2F-V). (b) The change in the OCP of p(C2F-V) after it was held at a −0.7 V vs Ag/AgCl for 300 s in PBS under air and N_2_ conditions. Due to the rapid drop of current values in air, it was not possible to monitor the ORR currents of p(C2F-V) using RDE and RRDE techniques. (c) The stability of OECT channel currents (normalized by the 10 s data). The devices were monitored first in N_2_ and then in air for 600 s. V_GS_ = 0.5 V and V_DS_ = 0.5 V. (d) Deconvoluted and peak-assigned high-resolution C 1s, O 1s, and N 1s XPS spectra of p(C2F-V).

The DFT calculations and our experimental results suggest that O_2_ interactions with reduced polymers are not solely governed by their LUMO levels but are influenced by the specific chemical moieties in each polymer. In fact, even polymers with deeper LUMOs, such as p(C2F-V)^39^ (4.56 eV), P-75^13^ (4.85 eV), and PBFDO^40−42^ (5.18 eV) (see chemical structures in Figure 5a and Figure S11) undergo ORR. As shown in Figures S12a-b and S13a-b, both P-75 and PBFDO facilitate ORR, generating mostly water as the reaction byproduct. Self-discharge experiments for all the films reveal their tendency to return to undoped states in air (Figure 5b, Figure S12c and S13c), similar to shallow LUMO polymers like P-90, p(C_6_-NDI-T), and BBL. These results confirm that deeper LUMOs do not necessarily inhibit ORR.

The next question is whether ORR affects the stability of the reduced films. By recording OECT channel currents for 10 min under O_2_-free and ambient conditions, we observed that P-90, p(C_6_-NDI-T), and BBL-based OECTs exhibited minimal current loss (<5% after 10 min) in both electrolyte environments (Figure 5c). In contrast, p(C2F-V) based OECT experienced a significant decrease in channel currents in the presence of O_2,_ despite being stable when operated under N_2_. Specifically, p(C2F-V) OECT currents decreased by 61.8% in air (Figure S14) despite the polymer’s deep LUMO.

DFT calculations and subsequent validation experiments suggest that a polymer-O_2_ complex can form (eq 2, (POO)^•–^), leading to charge localization/trapping, thus rendering the polymer site nonconducting. This complex can form either through weak physisorption of O_2_ or a much stronger bond. In the former case, removing O_2_ from the device environment restores the semiconductor’s conductivity.^43^ These sites can recover if bound O_2_ has a higher affinity for reacting with H^+^ (eqs 3-6). This is the case for P-90 and p(C_6_-NDI-T), where the complexes are only intermediate. BBL, on the other hand, does not form such complexes. In the latter case (stronger polymer-O_2_ complex), the conductivity of site P is permanently lost, which can be the origin of O_2_-related degradation. The nature of such (*POO*)^•–^ complexes will depend on the polymer’s functional group to which O_2_ binds. We hypothesize that p(C2F-V) film degrades in air because it forms strong (POO)^•–^ complexes.

To test this hypothesis, we conducted DFT calculations. Table S3 shows that p(C2F-V) displays lower overpotentials and undergoes one electron reduction more easily compared to P-90, p(C_6_-NDI-T) and BBL, with less negative reduction potentials. The easier access of O_2_ to the one-electron reduced polymer can be attributed to the more electrophilic nature of p(C2F-V) inherent to the electron deficient lactone and lactam rings.^39^ The subsequent interactions of the polymers with O_2_ was analyzed by considering potential binding sites on the polymer backbone. Unlike P-90 and p(C_6_-NDI-T), where O_2_ binds to the thiophene unit, O_2_ interacts with the lactam ring in p(C2F-V) and cleaves the amide bond through a ring opening mechanism (see Figure S15 for the chemical structures after the ring opening). The corresponding O_2_-bound complex is positioned at −1.2 eV for p(C2F-V) which is below the O_2_-free isomer and −2.5 eV below the O_2_-bound isomer when O_2_ is bound at the terminal alkene (Table S3). These energies also suggest the preference of p(C2F-V) for the O_2_-bound isomer compared to the O_2_-free one. Note again that the terminal alkene position is included solely because of the dimer model and does not represent the actual system; only the internal positions reflect the real system.

The loss of conductivity of p(C2F-V) during doping in air can thus be contributed by the concomitant loss of planarity due to the lactam ring opening. This additional flexibility in the lactam unit reduces the polymers’ rigid structure and disrupts the conjugation essential for electron transport. XPS provides evidence for the molecular changes in this deep LUMO polymer once it is electrochemically cycled in air (Figure 5d). Deconvoluting the peaks in the O 1s and C 1s spectra of p(C2F-V), we find that certain peak contributions change after reduction (Table S4). In Table 2, we summarize that the ratio of O between the carbonyl (O—(C=O)—C) and the alkoxy groups of the lactone (O—(C=O)—C) remains the same (although the percentage distribution of these bonds changes), suggesting that no change occurs in the lactone group. In contrast, the ratio of the ester-associated carbonyls from the lactone group (O=C—N) to the amide-associated carbonyls from the lactam group (O—(C=O)—C) increases and this is attributed to the ring opening at the amide carbonyl. We also note an increase in oxygen in the C–O bonds due to the conversion of carbonyl groups of the amide groups to C–O groups, while there is an increase in the ratio of C–C, C=C with respect to C–N bonds due to the lactam opening.

**Table 2:** Area Ratios Derived from Deconvoluted and Peak-Assigned High-Resolution XPS Spectra^a^ of Different Bonds to Determine the Degradation Sites in the Chemical Structure for p(C2F-V) after Electrochemical Doping in Air

	observation	bond	pristine	O_2_ cycled
O 1s	lactone stability	O—(C=O)—C: O—(C=O)—C	1.42	1.43
	lactam carbonyl instability	O=C—N: O—(C=O)—C	5.79	4.75
C 1s	C–N decrease	C—N: C–C, C=C	1.36	0.97
	C=O decrease	C=O: C–O	1.18	0.76

a See Figure 5c.

## Discussion

The standard electrode potential for ORR and an n-type of OMIEC’s reduction under neutral pH is:
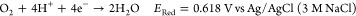
7
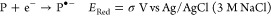
8

Where P is a site on the neutral polymer film, P^•–^ is the reduced site, and σ is the onset voltage for reduction. De Leeuw et al.,^7^ and Facchetti et al.,^8^ have argued that the reduction onset, σ, should be lower than *E*_*Red*_ of O_2_ for the n-type polymer to be ORR-inactive. When σ exceeds this value, the charged species reacts with O_2_. This reaction is as follows:
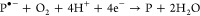
9

According to Koopman’s theorem, it can be assumed that *EA*^red^ ≈ −LUMO^Red^. It is also known that LUMO energies can be estimated from the reduction potentials as:^44^
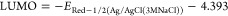
10

The standard potential for ORR (0.618 V vs *Ag*/*AgCl* (3 M NaCl)) corresponds to 5.011 eV according to eq 10. Therefore, from an energetic point of view, for the n-type OMIEC to be insensitive to O_2_, its LUMO should be higher than 5.011 eV.^44^ Nevertheless, empirical evidence from previous studies shows that an overpotential (η) of 0.5 to 1 V can exist,^7,8,45^ bringing the ORR potentials in the range of 0.118 V to −0.382 V vs Ag/AgCl (3 M NaCl). This, in turn, lightens the requirements on the LUMO level to the range of 4.511 eV (η = 0.5 V) and 4.011 eV (η = 1 V).^7,8^ Therefore, the >4 eV suggestion for O_2_-stability of polymers assumes an η of 1 V, i.e., an ORR onset of at least −0.382 V vs Ag/AgCl–if not more negative.

**Figure 6:**
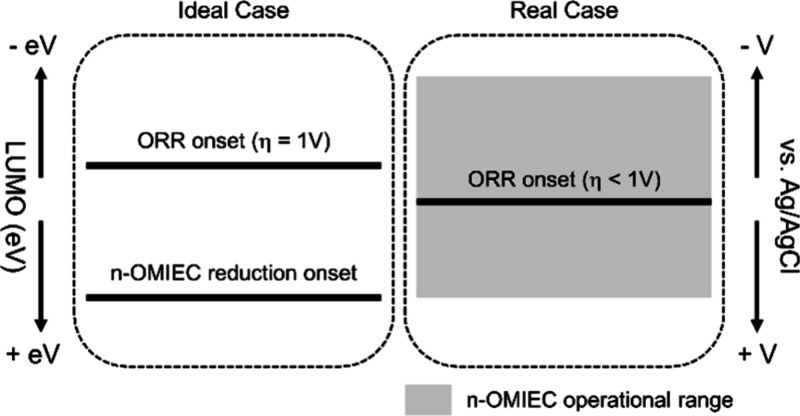
Illustration outlining the underlying cause of inevitable ORR in n-type OECTs. LUMO values are considered positive.

By comparing the LSV curves of our polymers under N_2_ and O_2_ conditions, we tested these assumptions. The ORR onset potentials of P-90, p(C_6_-NDI-T), and BBL are −0.16 V, −0.22 V, and −0.13 V vs Ag/AgCl, respectively, (Table S5) suggesting ORR overpotentials of 0.778, 0.838, and 0.748 V. Noble metals, characterized by their elevated electropositivity, require a substantial η to disrupt the stability of naturally adsorbed O_2_, facilitating the proton and electron transfers crucial for ORR.^46^ In contrast, OMIECs, with minimal electronegativity, merely require overpotentials to achieve conductivity, enabling electron transfer to O_2_. Consequently, ORR onset values for OMIECs closely align with their doping onset values, as illustrated in Figure 6. Therefore, the commonly cited “η = 1 V” does not universally apply and varies significantly among different polymers. Second, the notion that ORR in n-type OMIECs can be avoided solely by ensuring that the ORR onset is more negative than the reduction onset holds true only when the polymer operates precisely at its reduction onset. In practice, the polymer is often subjected to significantly more negative potentials than its reduction onset to ensure enough charge carriers are injected for effective current modulation. Therefore, despite their LUMO levels being deeper than 4 eV, all OMIECs evaluated in this study facilitated ORR, underscoring ORR inevitability during n-type device operation.

Furthermore, LUMO values that are slightly above or substantially higher than 4 eV do not reliably predict a polymer’s reactivity with O_2,_ as evidenced by the polymers tested here. This phenomenon mirrors findings in the OFET literature, where n-type polymers with higher LUMO values have not consistently demonstrated air stability.^12^ Additionally, lowering the LUMO level can be counterproductive when the goal is to attain a prebias OFF state in enhancement mode OECTs. As Figure S16 illustrates, p(C2F-V) and P-75 channels generate substantial currents at V_GS_ = 0 V due to the low threshold for doping, in contrast to the notably lower currents displayed by P-90, p(C_6_-NDI-T), and BBL under similar conditions.

In this work, polymers with shallower LUMO levels displayed minimal to no degradation, while a deeper LUMO one was susceptible to the irreversible formation of complexes with O_2_. While the exact mechanisms responsible for differences in ORR products are difficult to determine, it is clear that the influence of local chemical moieties becomes more significant once the ORR threshold is reached. Thus, the local chemical structure of n-type OMIECs plays a significant role in ensuring stable performance, provided that interactions with O_2_ are weak. However, other factors such as morphology, wetting, conductivity, and crystallinity can also affect ORR current levels and the resulting products.^47^ Therefore, it is crucial to consider a range of factors beyond just LUMO levels when evaluating the ORR capabilities and potential products of the semiconducting films.

## Conclusions

In this work, through electrochemical experiments and computational modeling, we studied the occurrence of ORR enabled by n-type OMIECs. We found that all n-type polymers undergoing reversible electrochemical reduction in water exhibit ORR, though the extent of ORR does not correlate with LUMO levels. Polymers with deeper LUMOs may exhibit limited ORR but are not suitable for enhancement mode OECTs due to high OFF currents and narrow operational window. Dynamic electrochemical and spectro-electrochemical measurements, combined with OECT performance evaluations under different environmental conditions, revealed that ORR can affect the stability of an n-type OMIEC even with a deep LUMO. ORR can either lead to film degradation (when the complex is trapped in a nonconductive state) or proceed to form H_2_O/H_2_O_2_ (enabled by the presence of H^+^ ions), converting the polymer back to its neutral state. We propose that the degradation of n-type OECTs is governed by the polymer-O_2_ bond strength versus the propensity of the formed complex to react with H^+^ and cannot be fully mitigated by tuning LUMO levels. Synthesizing BBL-like polymers with no binding site for O_2_ or P-90/p(C_6_-NDI-T)-like polymers with low O_2_ affinity sites, may be advantageous. Our findings suggest that evaluating the theoretical reactivity of functional groups toward O_2_ can aid in the synthesis of O_2_-resistant OMIECs, as illustrated by the case p(C2F-V). Moreover, molecules can be designed with functional groups that interact with O_2_ or H_2_O, but their reactivity can be minimized by introducing steric hindrances that render these sites inaccessible. Further collaboration between polymer chemists and theoreticians is necessary to identify specific moieties for developing polymers with enhanced stability and performance.

Our results have several practical implications. First, we demonstrated that while the OECT channel currents are unaffected by ORR, the gate currents increase due to ORR. In our specific case, increased I_GS_ does not disturb the electrochemical potential of the gate electrode because we used a nonpolarizable gate – Ag/AgCl (3 M NaCl), which maintains its potential via the Ag(s)/AgCl(s)/Cl^–^(aq) 3-phase equilibrium where chlorine ions (Cl^–^) are in saturation. However, nonpolarizability and infinite capacitance are theoretical ideals, and prolonged current extraction can alter the potential of a reference electrode, leading to inaccurate transfer characteristics. Moreover, in applications that rely on polarizable gate electrodes, high current withdrawals through increased I_GS_ due to ORR may shift the gate’s electrochemical potential, leading to inaccurate device outputs. Second, chemical analysis of the n-type OECT operational environment revealed that ORR products pose another challenge, as their accumulation may be toxic or interfere with the actual measurement. Although using a large electrolyte volume can mitigate this issue, it may not always be feasible, particularly in applications requiring small electrolyte volumes (e.g., sweat-based biosensing). This is especially concerning for long-term or chronic monitoring of cells or tissues, where there is no perfusion of fresh media or limited biological fluid flow. Third, our discharging experiments indicated that using current n-type OMIECs for charge storage or neuromorphic devices in ambient conditions remains challenging due to the rapid dedoping caused by ORR. Additionally, the example of BBL shows that the absence of O_2_ does not necessarily prevent self-discharge processes when other faradaic reactions (e.g., HER) occur. The fast discharging due to ORR presents a significant challenge for charge and memory storage technologies that utilize n-type OMIECs.

## Methods

### Materials

P-90, p(C_6_-NDI-T), P-75, p(C2F-V), and PBFDO were synthesized using existing protocols.^24,40,48,49^ BBL, methanesulfonic acid (MSA), and 1,1,1,3,3,3-Hexafluoro-2-propanol (HFIP) were purchased from Sigma-Aldrich and used as received.

### Polymer Solution Preparation

P-90, p(C_6_-NDI-T), and P-75 solutions were prepared by dissolving the respective polymer in chloroform at 5 mg/mL concentration. The solutions were kept on a 45 °C hot plate for 30 min before use. Five mg/mL BBL solution was prepared by dissolving the BBL powder in MSA and heating it at 90 °C for 1 h while stirring. p(C2F-V) solution (5 mg/mL) was prepared by dissolving the polymer in HFIP. PBFDO was acquired as dissolved in DMSO.

### Electrode and Resistor Preparation

Ten μL of P-90, p(C_6_-NDI-T), P-75, or p(C2F-V) solution was spin-coated on a removable glassy carbon disc electrode at 1200 rpm with 200 rpm of acceleration. The films were left to dry in an ambient environment for 30 min, after which they were blow-dried using an N_2_ gun. Discs were plasma-activated and coated using the same parameters for the BBL case. After coating, the disc was kept in DI water for 30 min before being vacuum-dried in an oven at 100 °C. Ten μL of PBFDO in DMSO was drop casted on the glassy carbon electrode and annealed for 5 h in a vacuum oven at 100 °C. Finally, the discs were loaded onto either a rotating disc or rotating ring disc electrode. Interdigitated electrodes (MicruX technologies, ED-IDE3-Au) were used for conductance experiments. The electrodes were cleaned with ethanol, IPA, and DI water while spinning on a spin coater and were then blow-dried using an N_2_ gun. The polymers were deposited as described above.

### Electrochemical Measurements

A VSP-300 Biologic potentiostat/galvanostat was used to record the cyclic and linear sweep voltammograms. All measurements were done in 1X PBS (pH = 7.4) with a polymer-coated glassy carbon rotating disc or the disc of a rotating ring disc electrode as the working electrode, Ag/AgCl (3 M NaCl) as the reference electrode, and a Pt wire inside a salt bridge as the counter electrode. Measurements were done in N_2_-rich and O_2_-rich electrolytes (PBS) by bubbling the respective gas for at least 30 min before measurements. A gas blanket was maintained above the electrolyte during the measurements to ensure stable conditions and avoid perturbation. For rotation control, a Gamry controller was used. For experiments where the O_2_ to N_2_ concentration of the bubbled gas was varied, we used the MXM Fusion Flow gas mixing system and the Fusion Flow computer software to control the O_2_ and N_2_ percentages throughout the experiment.

For rotating ring disc electrode (RRDE) experiments, the ring electrode was set at 0.7 V vs Ag/AgCl throughout the measurements for detecting the H_2_O_2_ generated at the disc. All measurements using the RRDE were done at a 1200 rpm rotation speed. RRDE measurements were also done in pH 3.3 (HCl) acidic and pH 13 (KOH) basic media with the same potential and environmental conditions mentioned above. An established analytical model determines the ORR pathway by measuring the effective number of electrons participating during ORR across the scanned voltage range.^50^ According to the model, the apparent electron transfer number (*n*), otherwise known as the average number of electrons consumed by each O_2_ molecule, is given by:
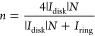
11where *I*_disk_ is the disk current, *N* is the collection efficiency of the ring electrode (measured as 0.25 using the process described by the manufacturer), and *I*_ring_ is the current generated by the ring electrode. *I*_ring_ arises from the oxidation of H_2_O_2_ hydrodynamically pushed from the disk electrode as it rotates. *N* determines how much of the H_2_O_2_ produced by the disk is oxidized by the ring electrode. An *n* value close to 2 means 100% reaction path (I), and *n* close to 4 signifies 100% reaction path (II).

### OECT Fabrication and Characterization

The OECTs were prepared using standard photolithography procedures. Briefly, 4-in. glass wafers were cleaned in piranha solution before coating with patterning photoresist and subsequently exposed using a custom photomask. Metal interconnects were deposited using magnetron sputtering of chromium, acting as an adhesion layer, followed by gold. Lift-off was achieved using appropriate solvents. Our insulation and patterning layers were deposited using chemical vapor deposition of Parylene C. A thick photoresist layer was deposited, baked, and patterned before revealing the openings for semiconductor patterning. Lastly, the patterns were opened using reactive-ion plasma etching. The resulting interdigitated channel had a width and length of 17,860 and 10 μm, respectively. OECT characterization was done using a dual-channel source-meter unit (Keithley 2602-A). A custom-written code controlled the unit through LabVIEW. Ag/AgCl (3 M NaCl, ALS Japan) was used as the gate electrode for all measurements to keep uniformity with the electrochemical measurements. The interdigitated OECT fabricated in-house was tested using a 1X PBS solution (pH = 7.4) as the electrolyte. A PDMS well contained the electrolyte, with the gate electrode immersed from the top to create a controlled environment. The OECTs were operated in either an N_2_-filled glovebox or an ambient environment.

### pH and H_2_O_2_ Monitoring

Optical microsensors from PreSens Precision Sensing GmbH (Germany) were used to monitor pH levels. The pH microsensor (model NTH-HP5) is a needle-type sensor with a measurement range of 5.5 to 8.5. The sensor was regularly calibrated and manipulated using a dedicated micromanipulator. The pH probe was positioned 1–2 mm above the n-OECT and immersed in an electrolyte solution with a volume of 1 mL. H_2_O_2_ levels were measured using an ISO-HPO-2 peroxide macro sensor manufactured by WPI Technologies. The sensor has a response time of <5 s (90%) and a detection limit between 100 nM and 100 μM. When the sensors were saturated, collected aliquots were diluted to measure the correct concentration.

### Computational Details

All calculations were performed at the DFT level using the range-separated hybrid functional ωB97x-D^51^ accounts for 22% Hartree–Fock (HF) exact exchange at short-range and 100% HF exact exchange at long-range, as implemented in the Gaussian16 suite. Geometry optimizations were performed in the gas phase with 6-31+G(d) basis set. The stationary nature of all structures was confirmed by analytic computation of their vibrational frequencies at 298.15 K. All frequencies below 50 cm^–1^ were replaced by 50 cm^–1^ when computing vibrational partition functions.

Single point energy calculations were carried out at ωB97x-D with 6-311++G(d,p) basis set. Solvent corrections in water (ε = 80) were added to the gas-phase geometries using SMD^52^ continuum solvation model. For all species, a factor of RT × ln(24.46) was added to account for the 1 atm to 1 M standard state change at 298.15 K. Standard reduction potentials was calculated from the computed free energies using SHE (*E*^0^ = 4.44 V) as the reference electrode.

We used dimer models of three polymers for all electronic structure calculations, where we truncated the polymer chains capped with H atoms. For P-90, we replaced the alkyl chains with ethyl-methyl ether to mimic the EG chains and to permit direct comparison with the p(C_6_-NDI-T) dimer model with only one less thiophene unit than the P-90 dimer model. Spin-state energy analysis gave a doublet POO^•–^, triplet POOH^–^, doublet PO^•-^, and singlet POH^–^ as ground states.

### LEIPS

All films were deposited on 100 nm sputter gold on silica substrates using a spin coater at 2000 rpm for 30 s and a 5 mg/mL solution in chloroform. Low energy inverse photoemission spectroscopy (LE-IPES) was conducted in a single UHV Scienta Omicron system at 10–9 mbar in a homemade system in the Bremsstrahlung isochromatic mode in the adjoining chamber consisting of a monoenergetic electron source (Staib) of 0.25 eV energy dispersion and drain current of 20–40 μA with a 2–3 mm sized electron spot, directed normal to the sample surface. The emitted IPES light was detected through a lens assembly (vacuum and airside), bandpass filter of 280 nm (4.43 eV), and solid-state photomultiplier tube (PMT) (Hamamatsu). Vacuum level calibration was made to the turn-on point in the drain current energy trace recorded simultaneously with the PMT signal, accounting for the bandpass energy. Note that all eV energies are reported with respect to vacuum.

### XPS

Samples were prepared by spin-coating polymers on Au-sputtered glass substrates with the parameters mentioned above. Electrochemical cycling was performed using cyclic voltammetry with respect to a leak-free Ag/AgCl reference electrode, and platinum was used as the counter electrode at 5 mV/s for 20 cycles between 0.4 and −0.8 V to obtain the cycled polymer film. All samples were stored in a N_2_-filled glovebox when not in use. XPS samples were prepared by spin coating polymers (5 mg/mL) at 1200 rpm with 200 rpm of acceleration for 30 s on a gold sputtered glass wafer substrate. XPS measurements were conducted using a Kratos Axis Supra system equipped with a monochromatic Al Kα X-ray source (*h*ν = 1486.6 eV). This source was operated at 150 W within an ultrahigh vacuum environment (in the range of ∼10^–9^ mbar). The spectra were recorded in a hybrid mode, utilizing electrostatic and magnetic lenses. Fixed S-5 analyzer pass energies of 80 and 20 eV were employed for acquiring survey and high-resolution spectra, respectively. Spectra calibration was performed using the carbon 1s peak at 284.8 eV as a reference. Deconvolution of the spectra was carried out using XPSPeak4 software, applying Gaussian and Lorentzian fitting methods, accompanied by Tougaard methods for background subtraction.

## Data Availability

All data needed to evaluate the conclusions in the paper are presented in the main test and/or the Supporting Information.

## Supporting Information Available

Extensive descriptions of the experimental and analytical methods, including detailed analyses of cyclic voltammograms, assessments using rotating disc and rotating ring-disc electrodes, organic electrochemical transistors, and density functional theory (PDF)
Transparent Peer Review report available (PDF)
