# Supplementary material for: Understanding Oxygen-Induced Reactions and Their Impact on n-Type Polymeric Mixed Conductor-Based Devices
Source: ACS Cent Sci. 2024 Nov 19;10(12):2229–41. doi: 10.1021/acscentsci.4c00654 (PMC11672553; doi:10.1021/acscentsci.4c00654)
Supplement: Supplementary file 1 — oc4c00654_si_001.pdf [file oc4c00654_si_001.pdf]

Supporting Information

Understanding Oxygen-Induced Reactions and their Impact on n-type Polymeric Mixed Conductor based Devices

Prem D. Nayak[1], Büsra Dereli[2], David Ohayon[1], Shofarul Wustoni[1], Tania Cecilia Hidalgo Castillo[1], Victor Druet[1], Yazhou Wang[1], Adel Hama[1], Craig Combe[3], Sophie Griggs[4], Maryam Alsufyani[3], Rajendar Sheelamanthula[3], Iain McCulloch[3,4], Luigi Cavallo[2], Sahika Inal[1*]

[1] Organic Bioelectronics Laboratory, Biological and Environmental Science and Engineering Division, King Abdullah University of Science and Technology (KAUST), Thuwal 23955-6900, Saudi Arabia

[2] Physical Sciences and Engineering Division, KAUST Catalysis Center, KAUST, Thuwal 23955-6900, Saudi Arabia

[3] KAUST Solar Center, Physical Sciences and Engineering Division, KAUST, Thuwal 23955-6900, Saudi Arabia

[4] Department of Chemistry, Chemistry Research Laboratory, University of Oxford, Oxford OX1 3TA, UK

[*]Corresponding Author: sahika.inal@kaust.edu.sa

Table S1. Energy levels of n-type OMIECs

Polymer	LUMO[a] (eV)	LUMO[b] (eV)
P-90	4.05	4.12
p(C_6-NDI-T)	[c]	4.23
BBL	4.02	4.30
P-75	-	4.85
p(C2F-V)	-	4.56

[a] measured by low energy inverse photoelectron spectroscopy (LEIPS); [b] measured using the reduction onset in cyclic voltammetry (CV) curve. CVs were carried out using a polymer-coated gold electrode, a Pt rod as a counter electrode, and Ag/Ag^+ as a reference electrode. The electrolyte was 0.1 M $TBAPF_6$ dissolved in acetonitrile. The Ferrocene/Ferrocenium (Fc/Fc^+) redox couple was used as a reference, and all potentials were reported versus this couple using 4.8 eV as ionization energy of Fc/Fc^+. [c] p(C_6-NDI-T) produced ununiform films, so the gold substrate influenced the LEIPS values. Repeated experiments led to varying LUMOs between 3.6-3.8, which is too shallow for this material, indicating patches of exposed gold substrate.

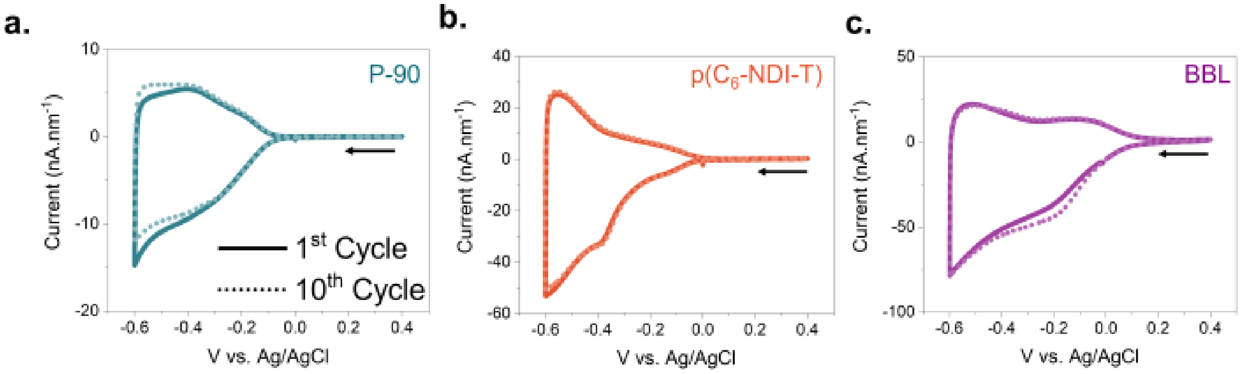

Figure S1. Cyclic voltammetry curves of **a)** P-90, **b)** p(C_6-NDI-T), and **c)** BBL film shown for first and tenth cycles recorded in PBS in air.

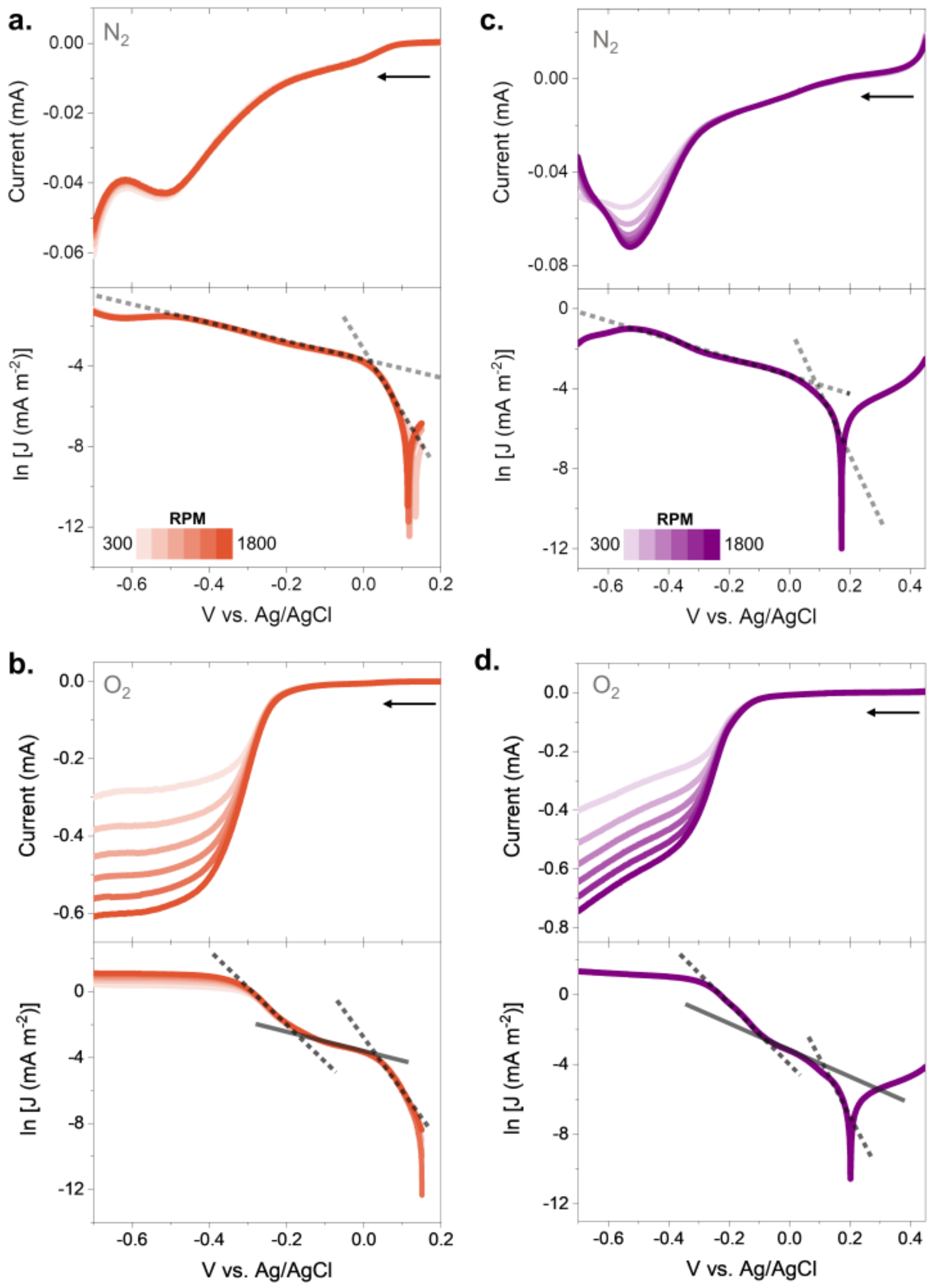

Figure S2. Linear sweep voltammograms for p(C_6-NDI-T) (left panel) and BBL (right panel) recorded in **a, c)** N_2-saturated and **b, d)** O_2-saturated PBS at different rotation speeds (300 to 1800 RPM) using a rotating disc electrode. Respective Tafel plots are shown in the bottom panel of each LSV curve. All scans were performed at 5 mV/s scan rate to ensure steady-state current collection. Arrows indicate the scan direction. Note that BBL undergoes HER from ~-0.45 V vs. Ag/AgCl onwards, evident from a slight increase in current upon rotation in N_2-saturated conditions (**c**).

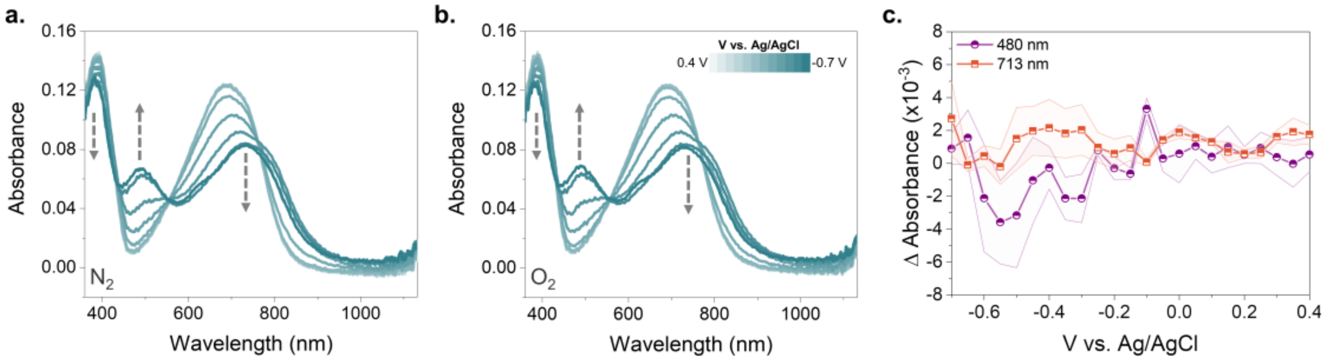

Figure S3. The evolution of P-90 absorption spectrum as a function of increasing doping potentials from 0.4 V to -0.7 V vs. Ag/AgCl applied through **a)** N_2-saturated PBS and **b)** O_2-saturated PBS; **c)** Absolute intensity differences between a selected peak (490 or 713 nm) recorded in O_2-saturated PBS and N_2-saturated PBS. All scans were performed at a 5 mV/s scan rate to ensure steady-state current collection. Error bars were calculated from more than 3 measurements performed on the same film.

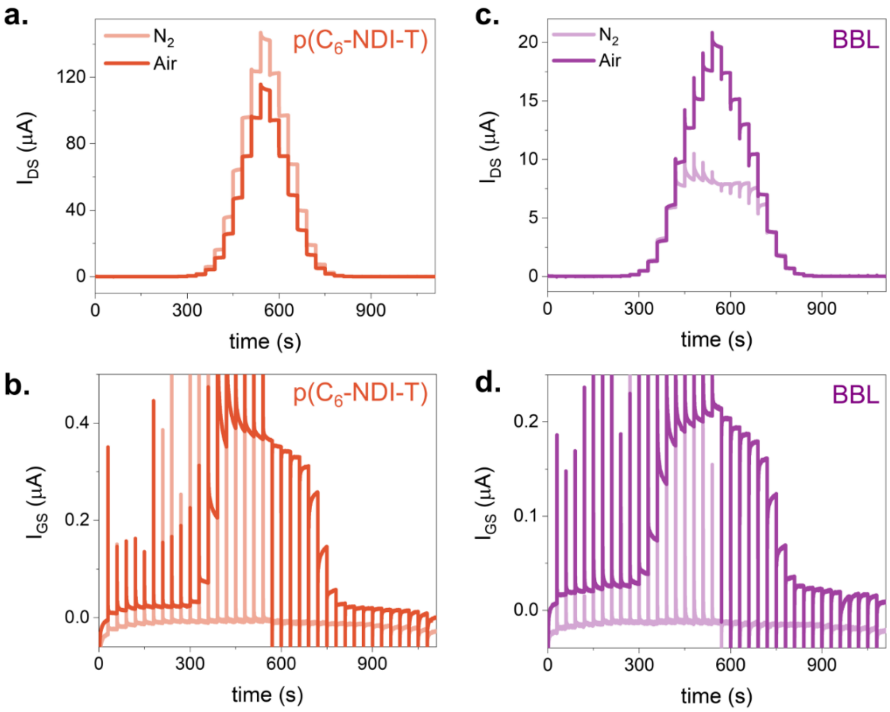

Figure S4. a, c) Drain currents and **b, d)** gate currents of p(C_6-NDI-T) (left) and BBL (right) OECTs recorded in an N_2-filled glove box and air. Measurements were taken during a gate-source voltage (V_{GS}) scan from -0.3 V to 0.6 V and back to -0.3 V while maintaining the drain-source voltage (V_{DS}) at 0.5 V. Each V_{GS} was maintained for 30 seconds. An Ag/AgCl reference electrode was used as the gate, and PBS as the electrolyte.

Section 1. Probing ORR products

ORR can occur via two main pathways:

$O_2 + 2H^+ + 2e^- \rightarrow H_2O_2$ (S-1)

$O_2 + 4H^+ + 4e^- \rightarrow 2H_2O$ (S-2)

ORR pathway was determined by calculating the effective number of electrons participating in ORR for each voltage at a rotation speed of 1200 rpm using an analytical model described elsewhere.[1,2] Based on this model, the apparent electron transfer number (n), which can be referred to as the average number of electrons consumed by each O_2 molecule, is given by:

$$n = \frac{4|I_{disk}|N}{|I_{disk}|N + I_{ring}} \quad \text{(equation S1)}$$

where I_{disk} is the disk current, N is the collection efficiency of the ring electrode (measured as 0.25 using the process described by the manufacturer), and I_{ring} is the current generated by the ring electrode. I_{ring} arises from the oxidation of H_2O_2 hydrodynamically pushed from the disk electrode as it rotates. N determines how much of the H_2O_2 produced by the disk is oxidized by the ring electrode. An n value close to 2 means 100% reaction path (I), and n close to 4 signifies 100% reaction path (II).[1]

Valid values of n lie between 2 and 4, where n = 2 signifies 100% H_2O_2 formation, and n = 4 corresponds to 100% H_2O formation. Finally, any n values between 2 and 4 correspond to a mixture of H_2O and H_2O_2 formation. We plot a heat map of such values under different pH (acidic –3.3, neutral –7.4, and basic –13.0) in **Figure S5**. We test the current response in different pH because if the reaction proceeds via the four-electron pathway, we expect a strong pH dependence.[3] At neutral pH, P-90 (n = 3.71) produces the highest proportion of H_2O out of the OMIEC series, whereas p(C_6-NDI-T) (n = 3.21) and BBL (n = 3.21) have a mixed production of H_2O and H_2O_2. In acidic conditions, all tested polymers produce much more H_2O than H_2O_2 (n > 3.74). In basic conditions, p(C_6-NDI-T) and BBL maintain a mixed pathway (n = 3.06), and H_2O_2 production is favored for P-90 (n = 2.79). The stronger pH dependence of P-90 compared to other polymers indicates that the ORR undergoes a four-electron preferential pathway.

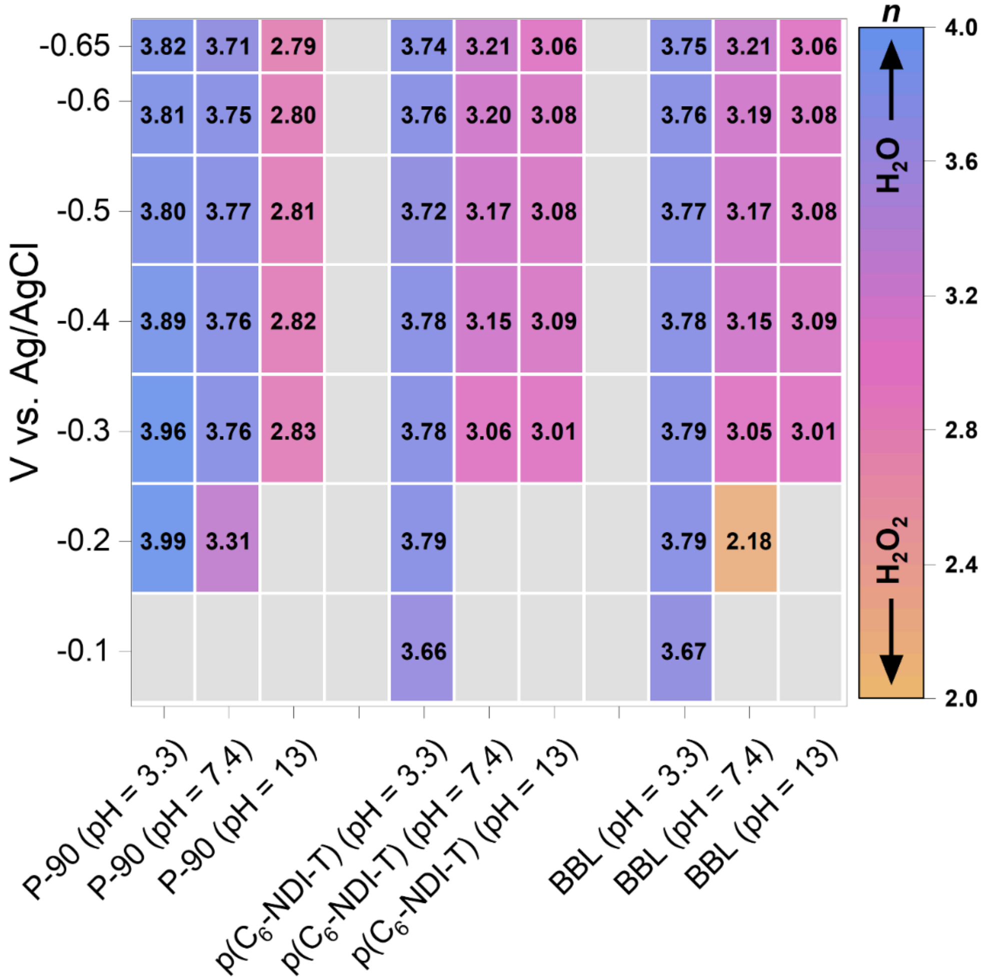

Figure S5. The potential-dependent heat map presents the number of electrons (*n*) used to reduce each O_2 molecule during LSV in different pH conditions. The data were recorded for polymer-coated glassy carbon electrodes rotated at 1200 rpm. The scan rate was 5 mV/s.

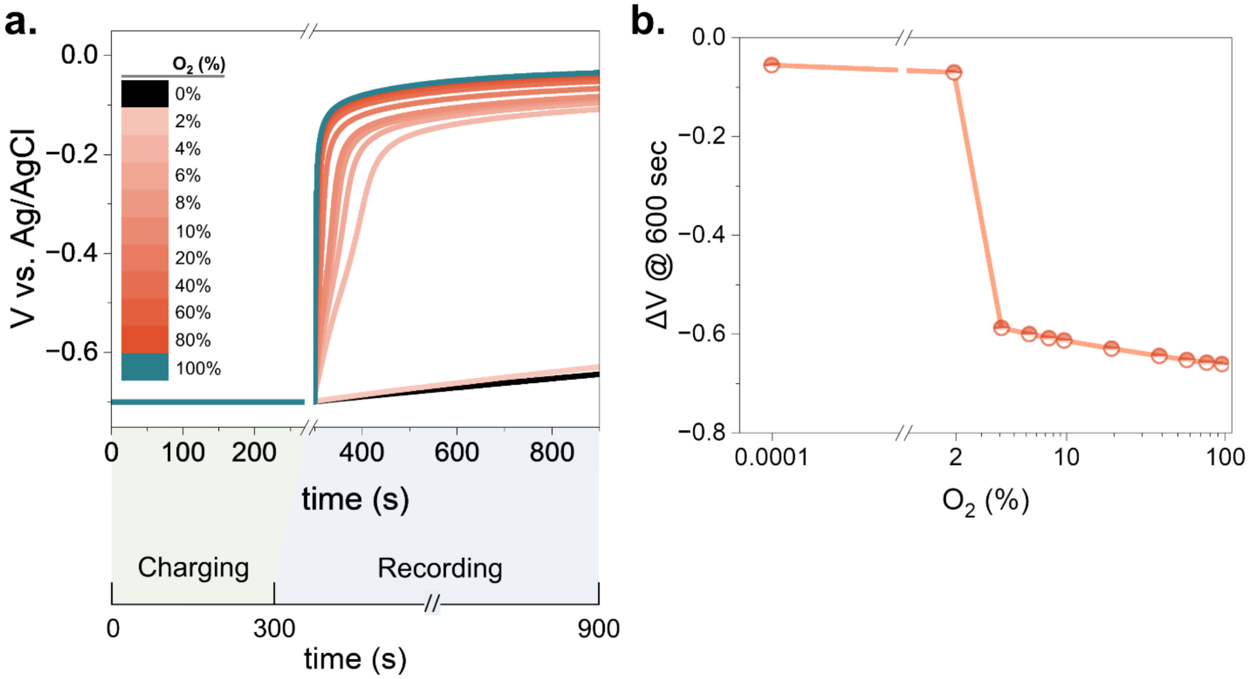

Figure S6. a) The change in the OCP of the p(C6-NDI-T) electrode after being held at -0.7 V vs. Ag/AgCl for 300 seconds in PBS under varying O_2 concentrations from 0 to 100%. **b)** The change in OCP 600 seconds after being held at -0.7 V vs. Ag/AgCl as a function of O_2 percentage.

Table S2. Predicted one-electron reduction potential of trimer models, and DFT calculated binding energy of molecular O_2 at the terminal position.

Reduction Reaction	E^0 vs. SHE (V)	Reduced film	O_2 binding energy (eV)
^{1}P-90 + e$^-$ → ^{2}P-90$^{\bullet -}$	-0.46	^{2}P-90$^{\bullet -}$	0.61
^{1}p(C_6-NDI-T) + e$^-$ → ^{2}p(C_6-NDI-T)$^{\bullet -}$	-0.63	^{2}p(C_6-NDI-T)$^{\bullet -}$	0.62
^{1}BBL + e$^-$ → ^{2}BBL$^{\bullet -}$	-0.49	^{2}BBL$^{\bullet -}$	1.56
$^3O_2$ + e$^-$ → $^2O_2^{\bullet -}$	-0.53		

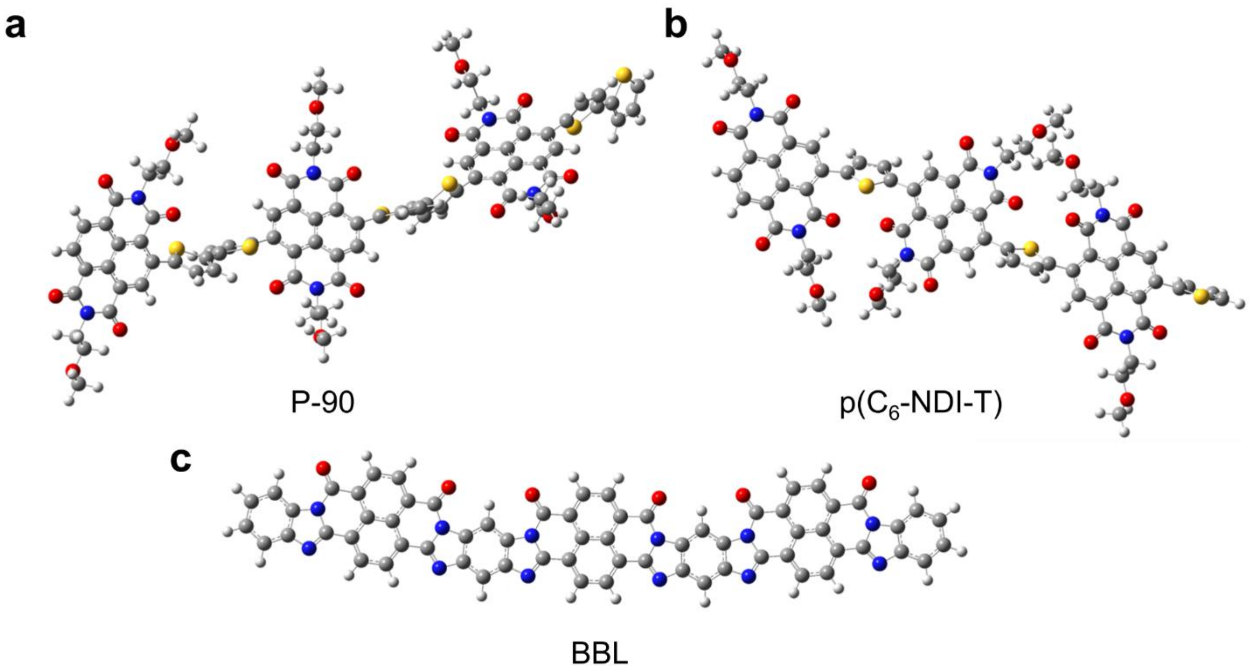

Figure S7. Trimer models of **a)** P-90, **b)** p(C6-NDI-T), **c)** BBL.

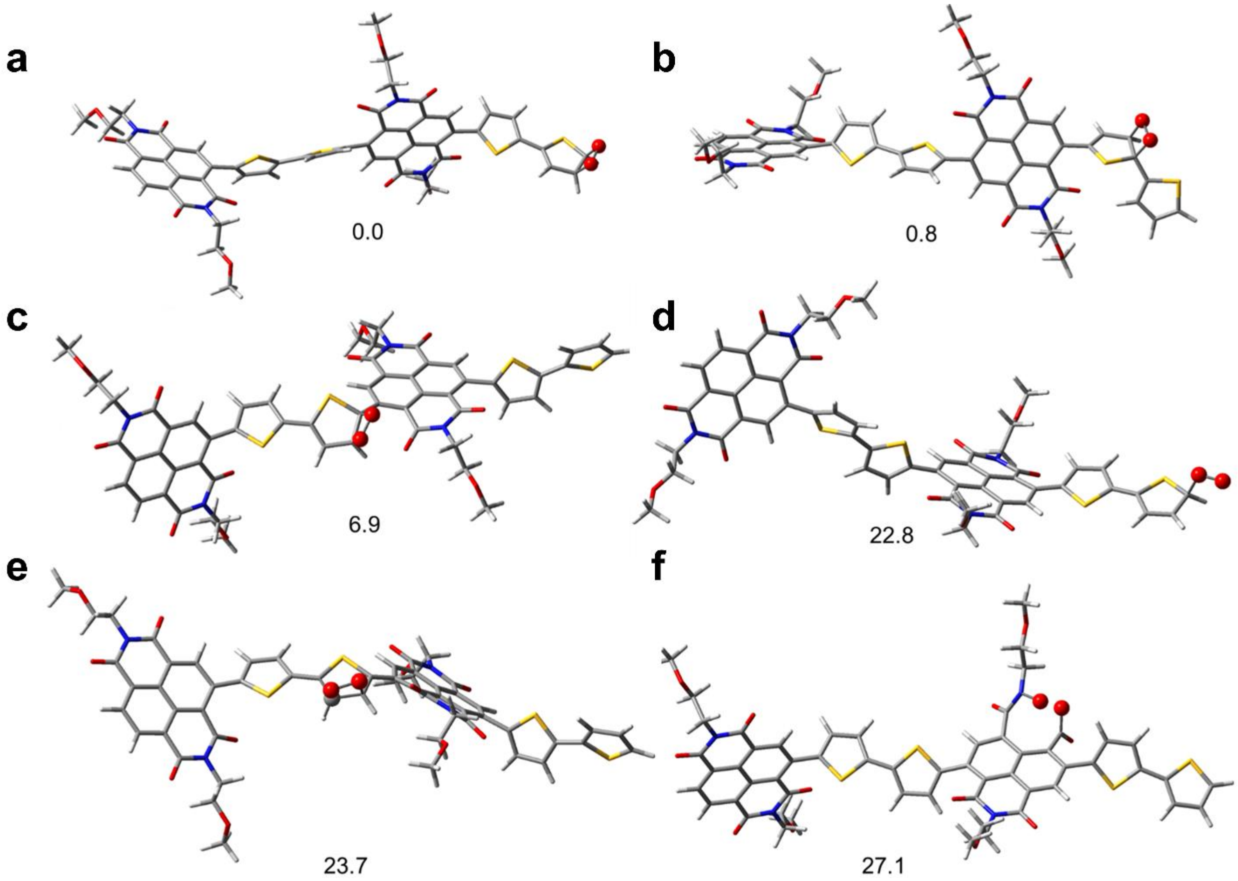

Figure S8. Relative free energies (in kcal/mol) for O_2 binding mode of $POO^{\bullet-}$ species for P-90 dimer. Note that the terminal alkene positions (in both end-on and monodentate modes) do not reflect the real system and are artifacts of the dimer model.

Section 2. Experimental validation of DFT results: Interactions between O_2 and dry OMIEC films

DFT studies suggest that while P-90 and p(C_6-NDI-T) allow O_2 adsorption to form a $POO^{\bullet-}$ like species (Equation **2**). Since $POO^{\bullet-}$ will hold a localized non-conducting electron, we hypothesize that without H^+ ions, $POO^{\bullet-}$ will not be converted back to P or $P^{\bullet-}$ (**equations 3-6**), and these films will lose their conductive state. On the other hand, according to DFT, no obvious binding sites in the BBL backbone exist. BBL instead enables the tunneling of electrons to O_2 (i.e., $P^{\bullet-}$ directly transfers the electron to O_2 to form P). If these mechanisms are correct, we expect to 1) record lower currents for dry P-90 and p(C_6-NDI-T) films in air compared to N_2, and 2) the dry BBL film should exhibit the same current values in N_2 and air conditions.

To test this hypothesis, we performed sequential step-chronoamperometry measurements in dry conditions (no PBS) to eliminate H^+ ions. **Figure S9** shows that dry P-90 and p(C_6-NDI-T) display higher currents in N_2 compared to ambient conditions, indicating that the films have higher currents if they do not interact with O_2. This result suggests that doping sites are blocked (generate

POO•⁻) due to O_2 in the absence of H^+. Recall that this was not the case in the presence of H^+ ions: I_{GS} was higher in air to re-dope the POO•⁻ sites released by H^+ ions (**Figure 2 e**).

Performing the same experiment for BBL, we detected higher currents in air (**Figure S9c**). We postulate that this is due to BBL getting doped by the ambient H_2O (moisture), which generates H^+ ions.[4] When moisture is removed from the air, from 66 % down to 0.5 % relative humidity (%RH), we observe a sharp decrease in the BBL current (**Figure S10**).

These experiments demonstrated that limiting O_2 interactions resulted in a substantial increase in currents generated by NDI-based polymers. Conversely, BBL exhibited an opposite behavior due to moisture doping in air, showing no signs of O_2-related de-doping. Thus, the data support the DFT calculations, which predicted the energetic favorability of P-90 and p(C_6-NDI-T) for O_2 interactions and the unfavorable nature of BBL in this regard. However, operation in aqueous media leads to a similar effect of O_2 in the OECT currents for all three polymers due to the compensating H^+ ions and counter electrode/gate currents.

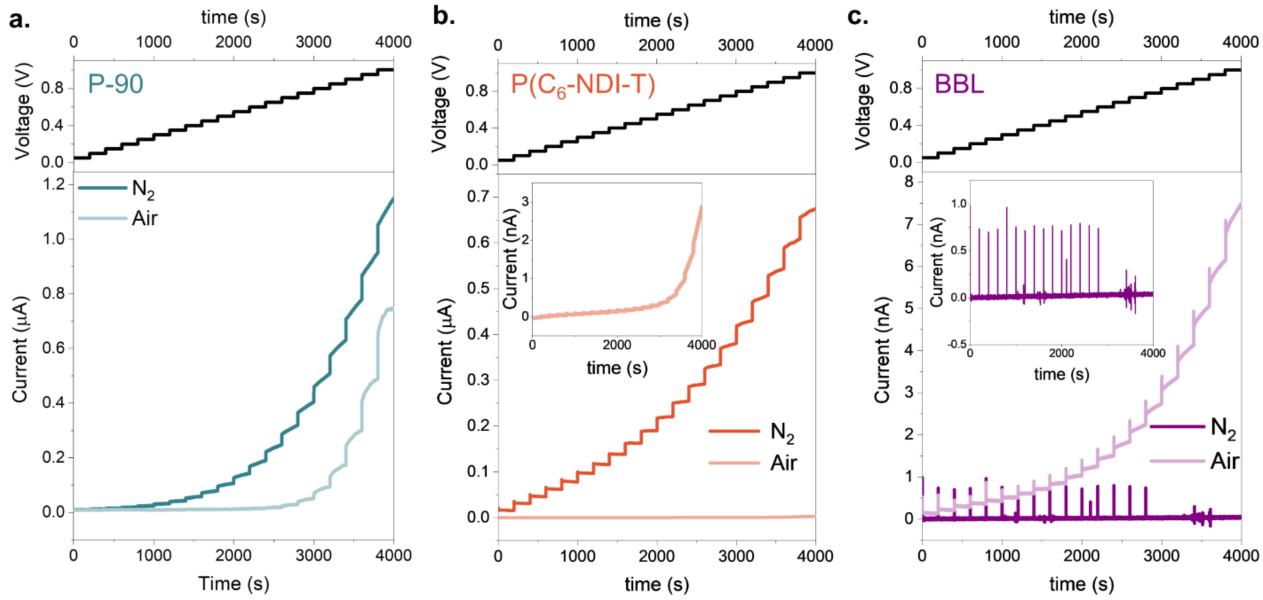

Figure S9. The current-time profile of dry n-type OMIEC films, deposited between the two terminals of an interdigitated electrode array (W=937144 µm, L=5 µm, 180 pairs), recorded in air and N_2-saturated conditions at different potentials. **a)** P-90, **b**) p(C_6-NDI-T), and **c)** BBL. Each voltage step was applied for 200 seconds from 0 V to 1 V with a step of 50 mV. The insets to **b)** and **c)** are zoomed-in currents recorded in air.

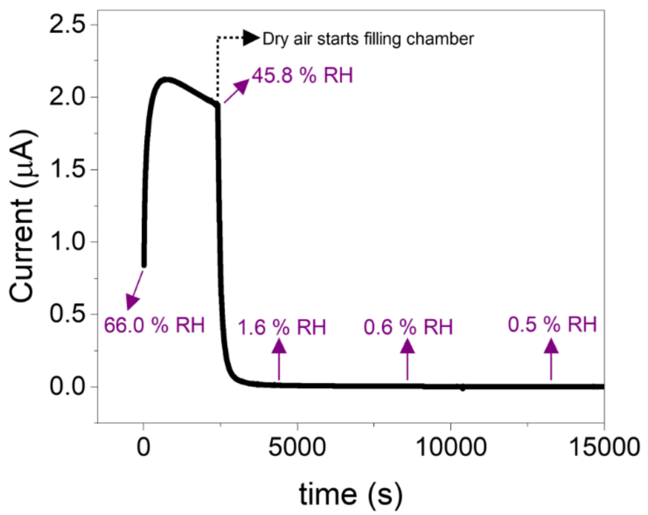

Figure S10. The BBL current over time with various levels of humidity in the chamber. The dashed arrow indicates the time point when we introduced dry air in the system, gradually removing the ambient moisture. The film was biased at 1 V.

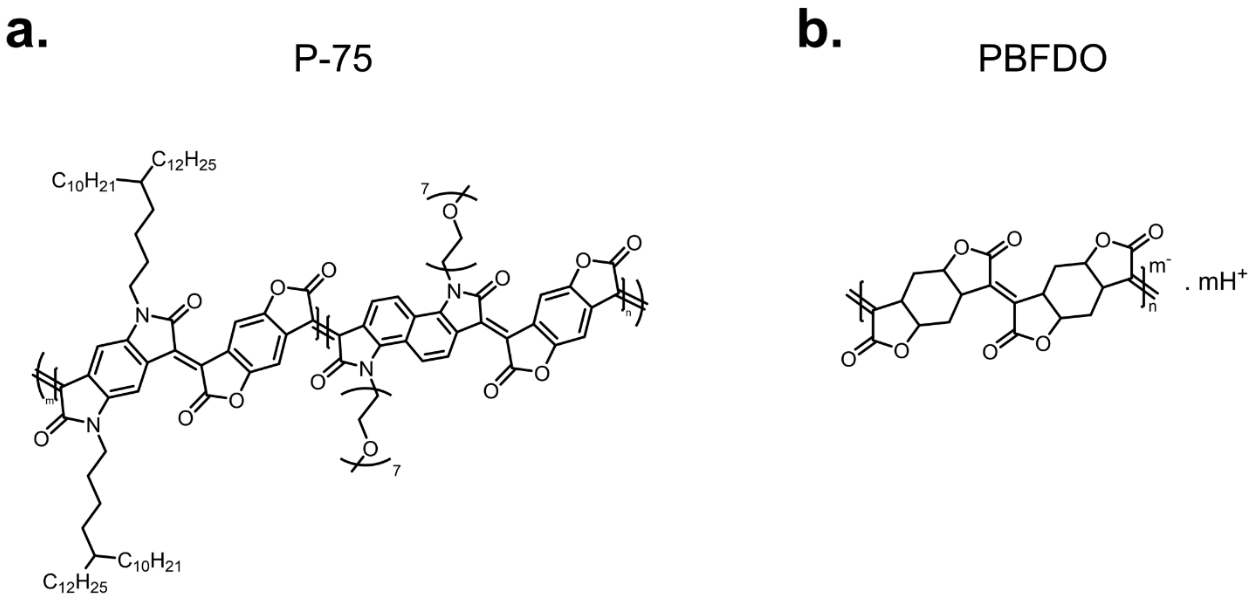

Figure S11. a) The chemical structures of the deep LUMO polymers P-75 (4.85 eV) and PBFDO (5.18 eV).

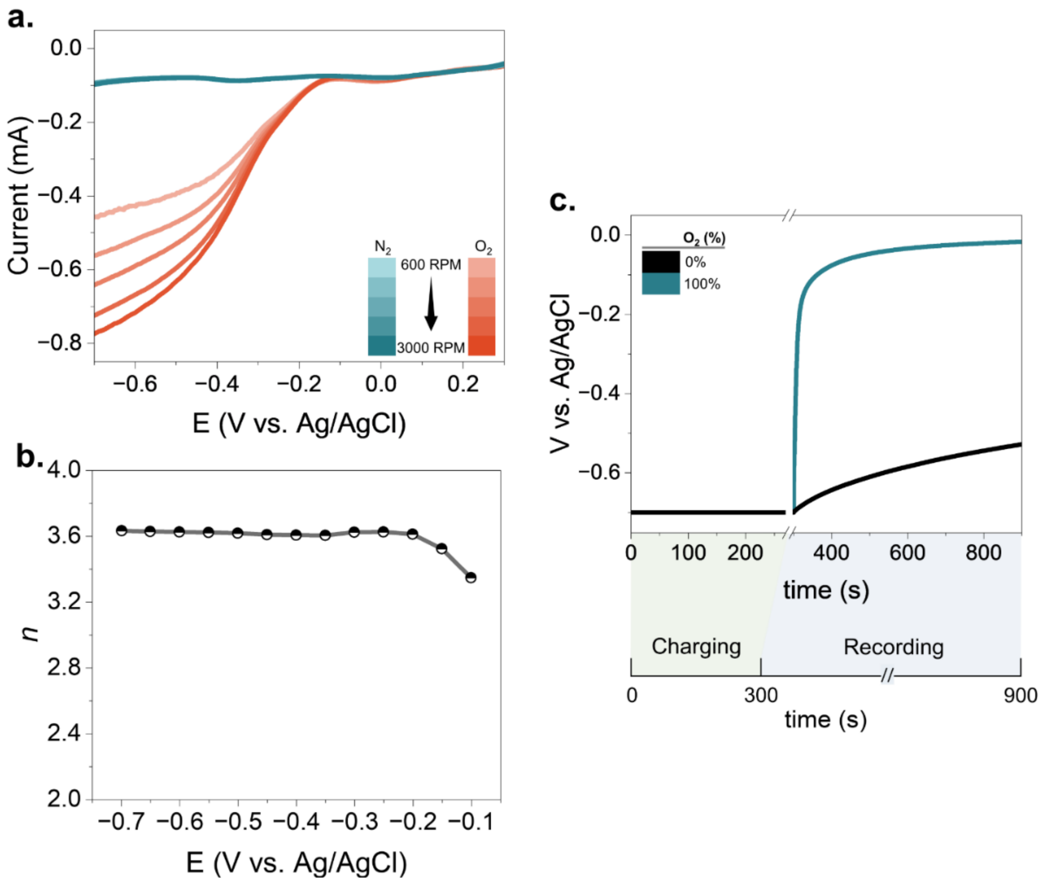

Figure S12. a) Linear sweep voltammograms for PBFDO in N_2- and O_2-saturated PBS at different rotation speeds (600 to 3000 RPM) using a rotating disc electrode. **b)** The number of electrons (n) used to reduce each O_2 molecule versus potential during LSV, recorded for polymer-coated glassy carbon electrodes rotated at 3000 RPM. **c)** The change in the OCP of PBFDO after being held at -0.7 V vs. Ag/AgCl for 300 seconds in PBS under O_2-saturated and N_2-saturated conditions. All scans were performed at a scan rate of 5 mV/s.

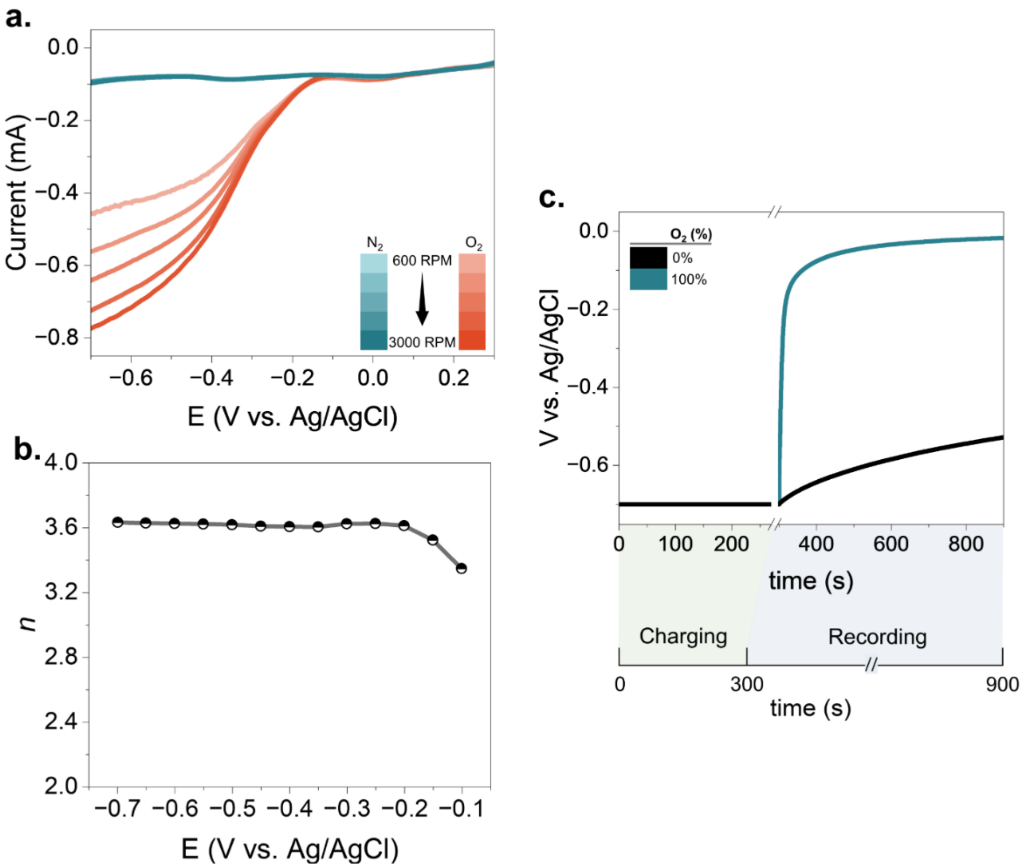

Figure S13. a) Linear sweep voltammograms for P-75 in N_2- and O_2-saturated PBS at different rotation speeds (600 to 3000 RPM) using a rotating disc electrode. **b)** The number of electrons (n) used to reduce each O_2 molecule versus potential during LSV, recorded for polymer-coated glassy carbon electrodes rotated at 3000 RPM. **c)** The change in the OCP of P-75 after being held at -0.7 V vs. Ag/AgCl for 300 seconds in PBS under O_2-saturated and N_2-saturated conditions. All scans were performed at a scan rate of 5 mV/s.

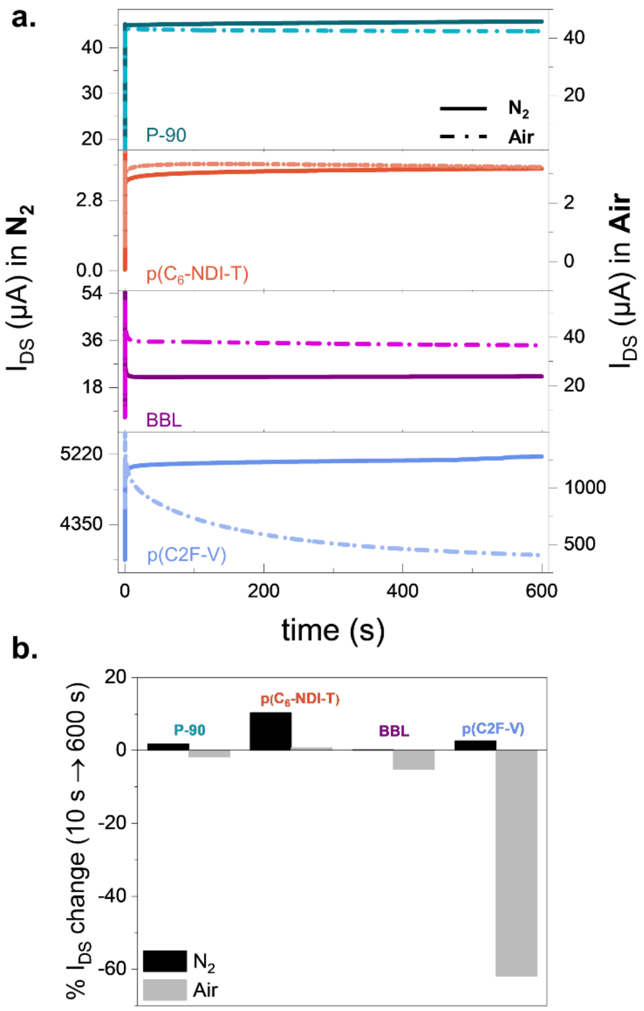

Figure S14. a) The evolution of OECT channel currents of the five polymers over time. The devices were monitored first in N_2 and then in air for 600 seconds. V_{GS} = 0.5 V and V_{DS} = 0.5 V. **b)** The percentage change in I_{DS} between t = 10 s and t= 600 s measured in N_2 and air. Negative values indicate a current drop over time.

Table S3. Predicted one-electron reduction potential of the molecular O_2 and the dimer model, and the DFT calculated binding energy of molecular O_2 at different positions of the isomer.

**Reduction Reaction**	**E^0 vs. SHE (V)**	**Reduced film**	**O_2 binding energy (eV)**	
			**Amide/lactam ring**	**Terminal alkene**
^{1}p(C2F-V) + e^- → ^{2} p(C2F-V)$^{\bullet -}$	-0.16	^{2}p-2CFV$^{\bullet -}$	- 1.20	1.33
$^3O_2 + e^- \rightarrow {}^2O_2^{\bullet -}$	-0.53			
^{1}P-90 + e^- → ^{2}P-90$^{\bullet -}$	-0.49			

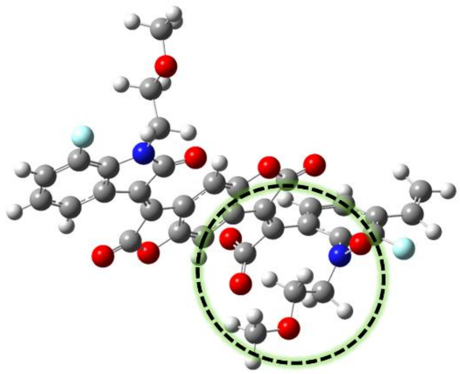

Figure S15. Predicted model of p(C2F-V) with cleaved amide bond due to O_2 interactions.

Table S4. Area values and percentages for different peak components derived from deconvoluted and peak-assigned high-resolution XPS spectra for p(C2F-V) before and after electrochemical cycling in ambient PBS.

Bond	Pristine	O_2 Cycled	Pristine	O_2 Cycled
**O 1s**	**Raw Area**		**% Area**	
O-(C=<u>O</u>)-C	3091.24	2800.71	9.64	8.54
O=C-N	17915.56	13307.82	55.89	40.58
O-C	8875.74	14722.53	27.69	44.90
<u>O</u>-(C=O)-C	2169.19	1955.10	6.76	5.96
**C 1s**				
C-C, C=C	8628.80	10402.71	24.60	30.33
C-N	11742.50	10012.67	33.48	29.20
C-O	6774.95	7882.30	19.31	22.98
C=O	7921.99	5991.08	22.59	17.47

Table S5. Threshold voltage of n-type OECTs operated in PBS with an Ag/AgCl gate and the ORR onsets observed from the LSV curves

Polymer	Threshold voltage (V vs. Ag/AgCl)	ORR onset (V vs. Ag/AgCl)
P-90	-0.18	-0.16
p(C_6-NDI-T)	-0.02	-0.22
BBL	0.16	-0.13

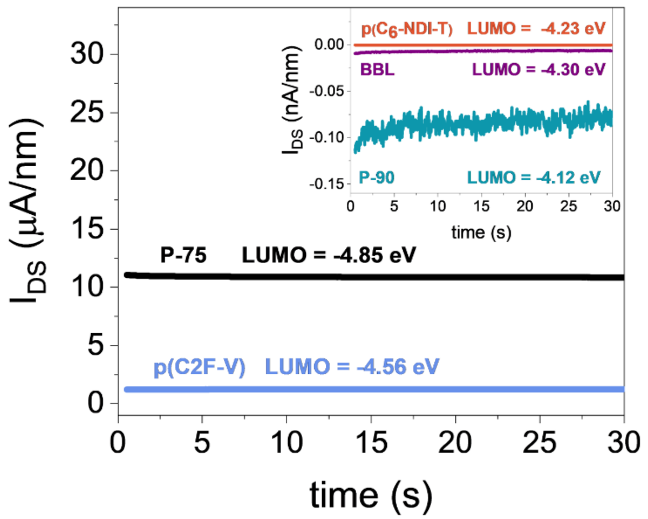

Figure S16. Thickness normalized OECT channel currents (I_{DS}) monitored at V_{GS} = 0 V and V_{DS} = 0.5 V for 30 seconds in PBS in N_2.

References

(1) Zhou, R.; Zheng, Y.; Jaroniec, M.; Qiao, S.-Z. Determination of the Electron Transfer Number for the Oxygen Reduction Reaction: From Theory to Experiment. *ACS Catal* **2016**, *6* (7), 4720–4728. https://doi.org/10.1021/acscatal.6b01581.

(2) Jia, Z.; Yin, G.; Zhang, J. Rotating Ring-Disk Electrode Method. In *Rotating Electrode Methods and Oxygen Reduction Electrocatalysts*; Elsevier B.V., 2014; pp 199–229. https://doi.org/10.1016/B978-0-444-63278-4.00006-9.

(3) Mitraka, E.; Gryszel, M.; Vagin, M.; Jafari, M. J.; Singh, A.; Warczak, M.; Mitrakas, M.; Berggren, M.; Ederth, T.; Zozoulenko, I.; Crispin, X.; Głowacki, E. D. Electrocatalytic Production of Hydrogen Peroxide with Poly(3,4-Ethylenedioxythiophene) Electrodes. *Adv Sustain Syst* **2019**, *3* (2), 1800110. https://doi.org/10.1002/adsu.201800110.

(4) Chen, Y.; Wu, H. Y.; Yang, C. Y.; Kolhe, N. B.; Jenekhe, S. A.; Liu, X.; Braun, S.; Fabiano, S.; Fahlman, M. In Situ Spectroscopic and Electrical Investigations of Ladder-Type Conjugated Polymers Doped with Alkali Metals. *Macromolecules* **2022**, *55* (16), 7294–7302. https://doi.org/10.1021/acs.macromol.2c01190.
