# Supplementary material for: Understanding Oxygen-Induced Reactions and Their Impact on n-Type Polymeric Mixed Conductor-Based Devices
Source: ACS Cent Sci. 2024 Nov 19;10(12):2229–41. doi: 10.1021/acscentsci.4c00654 (PMC11672553; doi:10.1021/acscentsci.4c00654)
Supplement: Supplementary file 2 — oc4c00654_si_002.pdf [file oc4c00654_si_002.pdf]

oc-2024-00654h.R1

Name: Peer Review Information for "Understanding Oxygen-Induced Reactions and their Impact on n-type Polymeric Mixed Conductor based Devices"

First Round of Reviewer Comments

Reviewer: 1

Comments to the Author

The paper describes an investigation of the ORR stability of n-type semiconducting polymers that are of interest for use in OECT, neuromorphic computing, and charge / energy storage applications. The investigation makes use of a wide range of chemical, electrochemical, and spectroscopic characterization techniques coupled with DFT calculations to explore the relationship between LUMO energy, chemical structure, and chemical reactivity relationships. The authors arrive at the general conclusion that chemical structure matters for ORR chemical reactivity, as opposed to just being dependent on the LUMO energy. From an (organic) physical chemistry standpoint, this is not a terribly surprising result; such relationships are readily reported across a diverse range of fields (e.g., in homogeneous and heterogeneous catalysis design, intercalation and conversion-based energy storage, etc.). To demonstrate broad interest, the authors should be able to make connections across these diverse chemistries to inform their own results. Several additional comments follow for the authors to consider in a revised version of the manuscript.

For the statement “a LUMO level deeper than ca. 4.0 eV”, what is the standard being referenced against? Given the broad reach of the chemical audience, it would be helpful to provide this information so that someone not deeply versed in the art can have a good guidepost.

The description of the results of the DiPietro work (Reference 14) is confusing. Chemical structure and electronic structure are intimately tied; at the level of quantum mechanics, everything can be described by wave functions. Are you rather trying to suggest that there may be regions of the structure that are more readily attacked (or not; e.g., steric blockers that prevent reaction) by oxygen being more important than an absolute energy level? If so, this needs to be made clear.

A difference of almost 0.2 eV across the LUMO energies is generally considered as a pretty significant change; indeed, many electrochemical studies strive to find stabilizations of LUMO by 200 meV. The authors need to better justify why 200 meV is not significant.

At what molar concentration of O2 does the presence of oxygen become a problem? The paper seems to report a zero O2 or fully saturated, but one might consider that there could be O2 concentrations where the effects are limited. It is suggested that O2 concentration variations be considered.

The DFT calculations need to be reconsidered. Using "a dimer model" necessarily sets up significant differences in the molecular structures and resulting orbitals being evaluated that will impact the evaluation of the results. This concept comes, thinking of a simple model, from different box lengths in the particle-in-a-box model. A dimer of p(C6-NDI-T) has 10 double bonds across the linear conjugated path, P90 has 28 (or, 14 if the structure as drawn in Figure 1 is considered as the dimer), and BBL has 12. While it is expected that the NDI-based polymers will have more localized LUMO due to chain twisting, the BBL is more planar and it is expected that the LUMO will be much more delocalized. It would be better for the authors to not choose dimers, but rather lengths of each system that allow for the effective conjugation length of the individual system to be reached. Such models will allow for more direct comparisons among these very different systems. This will also impact the predicted reduction potentials, and should hopefully provide a remedy to bring the DFT estimates more in line with experiment. (One note: the authors may want to consider models where, as best as they can, have an NDI more "central" in the oligomer chain. This will help to overcome potential issues arising from the asymmetric nature of the models as currently developed.)

How were the possible reaction points for O2 to the polymer backbone chosen? There is no explanation in the text. Why is it not suggested that the NDI may react? In the two NDI-containing polymers, this is where the LUMO will predominately reside; there will be very little wavefunction on the (bi)thiophene, and so it is not clear why O2 would react on these parts of the systems as drawn. It is also notable that the reactions are only depicted to be on the ends of the oligomers. Are the authors suggesting that O2 reactions will only take place at chain ends? Given the large number of repeat units (and the O2 saturation studies), it would be better to represent chemistries that take place in the middle of the representative oligomers.

Reviewer: 2

Comments to the Author

The manuscript investigates the issue of oxygen reduction reaction (ORR) affecting the performance and stability of n-type organic mixed ionic-electronic conductors (OMIECs). Specifically, the authors aim to understand how ORR impacts the charge storage performance and operational stability of these materials, which are essential for devices such as bioelectronic sensors, actuators, and soft charge storage systems.

Why is this research significant because n-type OMIECs are fundamental to various emerging technologies that interact with aqueous environments. The instability caused by ORR in these materials limits their practical applications, making it imperative to develop strategies to mitigate ORR and enhance device performance and durability.

The study focuses on three polymers: P-90, p(C6-NDI-T), and BBL. The authors used the rotating disc electrode (RDE) and linear voltammetry to measure ORR contributions on reduction currents in the polymers. In addition, the authors employed density functional theory (DFT) calculations and X-ray photoelectron spectroscopy (XPS) to analyze the interactions between the polymers and oxygen. Furthermore

the authors assessed the performance of organic electrochemical transistors (OECTs) using these polymers under different environmental conditions.

There are some very interesting conclusions.

1. Limited Correlation Between LUMO Levels and ORR: The study found a limited correlation between the lowest unoccupied molecular orbital (LUMO) levels of the polymers and ORR currents, indicating that simply lowering LUMO levels is not sufficient to mitigate ORR.

2. Chemical Moieties' Role: The backbone chemistry of the polymers plays a critical role in controlling O2-related degradation pathways and performance losses.

ORR and Device Functionality: ORR affects the stability and functionality of devices, particularly impacting gate currents without hindering channel currents in OECTs.

3. Charge Storage Challenges: Current n-type OMIECs are challenged by fast discharging due to ORR, making them inefficient for charge storage and neuromorphic devices.

Overall , the study is well planned and executed. The conclusion are well supported with experimental data. A deep understanding of the role of oxygen has been achieved. Here are a couple of minor suggestions.

1. To support conclusion 1, the author can have short discussion on n-PBDF, which has a much deeper LUMO than the listed three polymers and also interaction with oxygen and water. Oxygen dedopes n-PBDF, generating water, while water redopes the polymer and forms hydrogen peroxide. It is a dynamic process.

2. For conclusion 3, charging and discharging are intrinsically asymmetrical in conducting polymer, more generally, in redox polymers. Based on the needs, it can be altered. If bistability is truly desired, it can be accomplished through the selection of electrolyte and the electrode modification. Of course, it is difficult to realize in biosensors which have to function in the biological environment.

Author's Response to Peer Review Comments:

Dear Editor and reviewers,

We sincerely appreciate the reviewers' valuable time and constructive comments, which have greatly helped us improve our manuscript (oc-2024-00654h). In response, we have revised our work by refining the experimental design, providing more effective descriptions of our existing results, and clarifying how our conclusions can be extended to other polymers. All changes and additions are highlighted in yellow both here and in the revised manuscript and supplementary files. We hope you find the updated version of our work ready for publication and look forward to your feedback.

On behalf of all authors,

Sahika Inal

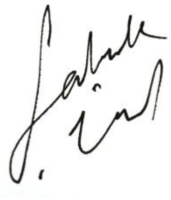

Reviewer 1:

The paper describes an investigation of the ORR stability of n-type semiconducting polymers that are of interest for use in OECT, neuromorphic computing, and charge / energy storage applications. The investigation makes use of a wide range of chemical, electrochemical, and spectroscopic characterization techniques coupled with DFT calculations to explore the relationship between LUMO energy, chemical structure, and chemical reactivity relationships. The authors arrive at the general conclusion that chemical structure matters for ORR chemical reactivity, as opposed to just being dependent on the LUMO energy. From an (organic) physical chemistry standpoint, this is not a terribly surprising result; such relationships are readily reported across a diverse range of fields (e.g., in homogeneous and heterogeneous catalysis design, intercalation and conversion-based energy storage, etc.). To demonstrate broad interest, the authors should be able to make connections across these diverse chemistries to inform their own results. Several additional comments follow for the authors to consider in a revised version of the manuscript.

We thank the reviewer for their valuable time spent reviewing our work and making recommendations. We agree with the reviewer that these results align with the established fields of homogeneous and heterogeneous catalysis design, as well as intercalation and conversion-based energy storage and we should note this and make connections to the broader chemistry audience.

We have added the following to our manuscript in the introduction section on Line 84:

"Furthermore, while the importance of molecular design—such as specific functional groups, stereochemistry, and the introduction of steric hindrances—on a material's chemical reactivity has

been well-established in the fields of organocatalysis[15–17] and intercalation-based energy storage devices,[18,19] these concepts have not been explored in detail for organic mixed conductors, which are susceptible to various faradaic reactions."

(15) Kiesewetter, M. K.; Shin, E. J.; Hedrick, J. L.; Waymouth, R. M. Organocatalysis: Opportunities and Challenges for Polymer Synthesis. *Macromolecules*. March 9, 2010, pp 2093–2107. https://doi.org/10.1021/ma9025948.

(16) Dalko, P. I.; Moisan, L. Enantioselective Organocatalysis. *Angewandte Chemie - International Edition*. October 15, 2001, pp 3726–3748. https://doi.org/10.1002/1521-3773(20011015)40:20<3726::AID-ANIE3726>3.0.CO;2-D.

(17) Melnyk, N.; Garcia, M. R.; Iribarren, I.; Trujillo, C. Evolution of Design Approaches in Asymmetric Organocatalysis over the Last Decade. *Tetrahedron Chem*. Elsevier Ltd March 1, 2023. https://doi.org/10.1016/j.tchem.2023.100035.

(18) Barbosa, J. C.; Fidalgo-Marijuan, A.; Dias, J. C.; Gonçalves, R.; Salado, M.; Costa, C. M.; Lanceros-Méndez, S. Molecular Design of Functional Polymers for Organic Radical Batteries. *Energy Storage Mater* **2023**, *60*. https://doi.org/10.1016/j.ensm.2023.102841.

(19) Lu, Y.; Zhang, Q.; Li, L.; Niu, Z.; Chen, J. Design Strategies toward Enhancing the Performance of Organic Electrode Materials in Metal-Ion Batteries. *Chem*. Elsevier Inc. December 13, 2018, pp 2786–2813. https://doi.org/10.1016/j.chempr.2018.09.005.

For the statement "a LUMO level deeper than ca. 4.0 eV", what is the standard being referenced against? Given the broad reach of the chemical audience, it would be helpful to provide this information so that someone not deeply versed in the art can have a good guidepost.

We thank the reviewer for pointing this out. We have updated the manuscript to clarify that the 4.0 eV value and all-electron energy values are referenced to the vacuum energy level, which is assumed to be at 0 eV. In the first instance (Line 59), we added:

"Consequently, it has been proposed that n-type materials with a LUMO level deeper than ca. 4.0 eV (with respect to vacuum) are necessary for stable OFET operation."

Additionally, we added:

"Note that all eV energies are reported with respect to vacuum" in the methods section describing the LEIPS measurements for LUMO energy determination (Line 655).

The description of the results of the DiPietro work (Reference 14) is confusing. Chemical structure and electronic structure are intimately tied; at the level of quantum mechanics, everything can be described by wave functions. Are you rather trying to suggest that there may be regions of the structure that are more readily attacked (or not; e.g., steric blockers that prevent reaction) by oxygen being more important than an absolute energy level? If so, this needs to be made clear.

We thank the reviewer for pointing out this important point. Indeed, chemical and electronic structures are intimately tied. DiPietro noted the following in his work (which, our results support):

1. The interaction with reactants occurs stepwise, making it crucial to understand these processes.
2. Reducing these complexities to a single energetic value (i.e., the LUMO) oversimplifies the interactions and different energetics of various reactions.
3. Energetics that define the propensities of different parts of the polymer to react with O_2 can and do vary (Figure S8).

A good point raised by the reviewer is that once the propensities of different parts of the molecular structure to react with O_2 and H_2O are determined, chemists can design polymers such that steric hindrances can be introduced in high-propensity locations to prevent/retard such reactions. They can better identify and understand the groups within the polymer that are less likely to engage in such reactions. This approach allows for a more nuanced understanding of polymer stability and the development of materials better suited to resist degradation by environmental factors.

To describe better what is mentioned above, we have modified the text about Di Pietro work and added the following in line 76:

"Di Pietro et al. highlighted the importance of considering the energetics of specific chemical sites within polymer structures and examining their interactions with O_2 and H_2O_2, rather than focusing solely on LUMO.[14] They critiqued the conventional approach of simplifying polymer air stability to a single value representing the polaron energy on the polymer chain, with its onset defined by the LUMO."

To highlight the possibility of introducing steric hindrances, we have added the following in Line 491 in the conclusion;

"Moreover, molecules can be designed with functional groups that interact with O_2 or H_2O, but their reactivity can be minimized by introducing steric hindrances that render these sites inaccessible."

At what molar concentration of O_2 does the presence of oxygen become a problem? The paper seems to report a zero O_2 or fully saturated, but one might consider that there could be O_2 concentrations where the effects are limited. It is suggested that O_2 concentration variations be considered.

Using one of our model systems, p(C_6-NDI-T), we investigated the O_2 concentration at which its presence becomes significant for polymer's charging performance. We repeated the electrochemical self-discharge experiments shown in **Figure 3**, but instead of using only two conditions (no O_2 with N_2 bubbling and saturated O_2 with O_2 bubbling), we used a gas mixer to vary the O_2 to N_2 ratio in the bubbled gas. The effects of O_2 began to appear at ca. 4% O_2 concentration, as shown in **Figure S6**. Given that air contains 21% O_2 and the solubility of O_2 in water is double that of N_2,[7] ambient water likely contains more than 21% O_2 (% in air), far exceeding the 4% O_2 threshold observed in our experiments.

We have added a figure in the Supplementary information showing the results of the experiments described above as **Figure S6**.

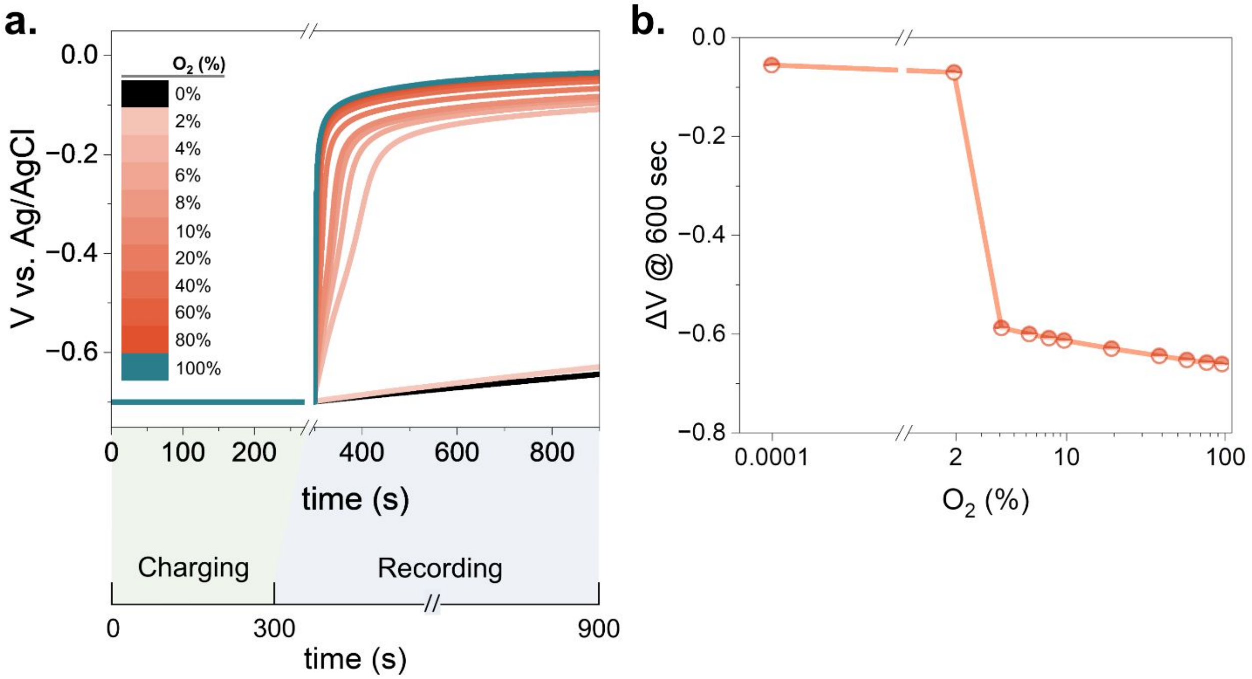

Figure S6. **a)** The change in the OCP of a p(C_6-NDI-T) electrode after being held at -0.7 V vs. Ag/AgCl for 300 seconds in PBS under varying O_2 concentrations from 0 to 100%. **b)** Change in the OCP of the electrode as a function of O_2 percentage in the environment.

We have also modified the main text as follows in line 276 to reflect the observations from the experiments:

"Furthermore, when we varied the O_2/N_2 ratio in the environment, we observed that O_2-driven self-discharge began at an O_2 concentration of 4%, much lower than the 21% O_2 present in air (**Figure S6**). Since O_2 is more soluble in water than in N_2,[37] the concentration of O_2 in ambient water is likely to be greater than 21%, allowing the O_2-related effects to manifest at lower ambient O_2 concentrations."

(37) Battino, R.; Seybold, P. G. The O 2/N 2 Ratio Gas Solubility Mystery. *J Chem Eng Data* **2011**, *56* (12), 5036–5044. https://doi.org/10.1021/je200878w

We also modified the "Electrochemical Measurements" methods section (line 585):

"For experiments where the O_2 to N_2 concentration of the bubbled gas was varied, we used the MXM Fusion Flow gas mixing system and the Fusion Flow computer software to control the O_2 and N_2 percentages throughout the experiment."

The DFT calculations need to be reconsidered. Using "a dimer model" necessarily sets up significant differences in the molecular structures and resulting orbitals being evaluated that will impact the evaluation of the results. This concept comes, thinking of a simple model, from different box lengths in the particle-in-a-box model. A dimer of p(C6-NDI-T) has 10

double bonds across the linear conjugated path, P90 has 28 (or, 14 if the structure as drawn in Figure 1 is considered as the dimer), and BBL has 12. While it is expected that the NDI-based polymers will have more localized LUMO due to chain twisting, the BBL is more planar and it is expected that the LUMO will be much more delocalized. It would be better for the authors to not choose dimers, but rather lengths of each system that allow for the effective conjugation length of the individual system to be reached. Such models will allow for more direct comparisons among these very different systems. This will also impact the predicted reduction potentials, and should hopefully provide a remedy to bring the DFT estimates more in line with experiment. (One note: the authors may want to consider models where, as best as they can, have an NDI more "central" in the oligomer chain. This will help to overcome potential issues arising from the asymmetric nature of the models as currently developed.)

A difference of almost 0.2 eV across the LUMO energies is generally considered as a pretty significant change; indeed, many electrochemical studies strive to find stabilizations of LUMO by 200 meV. The authors need to better justify why 200 meV is not significant.

We feel that both concerns expressed by the reviewer are correlated and therefore we try to address them together. We understand the reviewer's concern about the use of dimers. Unfortunately, we have been limited in our resources to consider much longer lengths of each system. However, we recalculated the redox potentials and energies of O_2 complexation for trimer models **(Figure S7)**. The predicted values are presented in **Table S2**. According to the computed values, the reduction potentials for trimer models remain nearly the same compared to the corresponding dimer models (**Table 1**). The most significant difference in the predicted reduction potentials observed between p(C_6-NDI-T) dimer (-0.54 V vs SHE) and trimer models (-0.63 V SHE) is around 90 mV which falls in the range of a reasonably accurate prediction using DFT methods. As for the O_2 binding energies, the variations between the two chain lengths seem negligible. Our results align with the seminal work of Zozoulenko and coworkers, where a dimer model for BBL was delineated with a charge well localized on LUMO and found to be sufficient for modeling n-doped BBL.[8,9] While the varying chain length may not change the charge localized region upon reduction, the secondary effects, such as ion-pairing, solvation, and the choice of density functional, might have a more pronounced effect on the predicted reduction potentials.[10,11] Further, the observed reduction potential (experimentally determined) significantly depends on the polymer morphology, wetting conditions, and experimental methods used, [12] which prevents achieving quantitative agreement even with a longer chain model. We hope that this answers the question about the 200 mV difference mentioned in the manuscript as well.

Furthermore, the results we achieved with the dimer model are sufficient to capture ORR trends we observed with the experiments. For example, our calculations point out the absence of O_2-bound intermediate due to an insurmountable barrier of 1.56 eV for BBL, compared to the barrier heights of 0.63 eV and 0.61 eV for P-90 and p(C_6-NDI-T), respectively, and these results were shown to be consistent experimentally (**Figure S9**). Finally, as we don't see significant change in any quantity predicted from dimer to trimer model, we can conclude that we are considering reliable conjugation lengths. Nevertheless, we hope to continue working with these systems in the future to better investigate which model is most suitable for each polymer, considering variations in molecular weights and conjugation lengths.

We added **Figure S7** in the supplementary information to show the predicted trimer models.

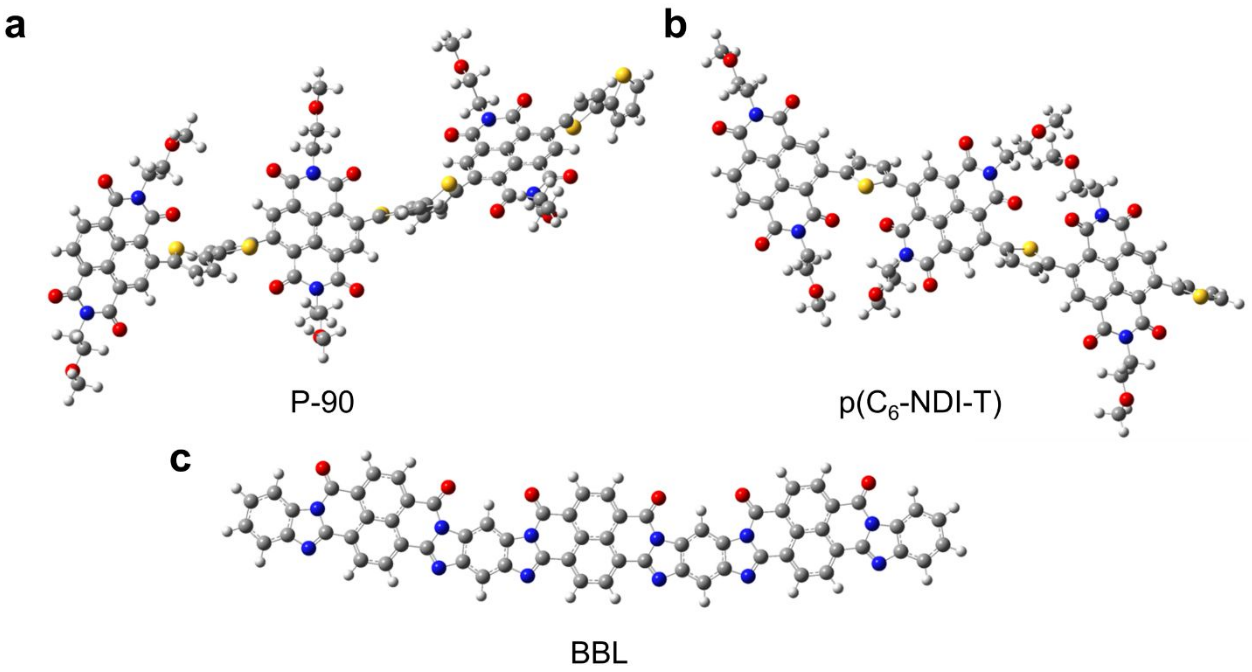

Figure S7. Trimer models of **a)** P-90, **b)** p(C6-NDI-T), **c)** BBL.

We also added the binding energies of O_2 to the predicted trimer models as **Table S2.**

Table S2. Predicted one-electron reduction potential of trimer models, and DFT calculated binding energy of molecular O_2 at terminal position.

**Reduction Reaction**	**E^0 vs. SHE (V)**	**Reduced film**	**O_2 binding energy (eV)**
^{1}P-90 + e^- $\rightarrow$ ^{2}P-90$^{\bullet -}$	-0.46	^{2}P-90$^{\bullet -}$	0.61
^{1}p(C_6-NDI-T) + e^- $\rightarrow$ ^{2}p(C_6-NDI-T)$^{\bullet -}$	-0.63	^{2}p(C_6-NDI-T)$^{\bullet -}$	0.62
^{1}BBL + e^- $\rightarrow$ ^{2}BBL$^{\bullet -}$	-0.49	^{2}BBL$^{\bullet -}$	1.56
$^3O_2$ + e^- $\rightarrow$ $^2O_2^{\bullet -}$	-0.53		

We added the following in line 280 to reflect the analysis done using the trimer model:
"When we conducted a similar analysis using a trimer model (**Table S2, Figure S7**), the predicted reduction potentials remained nearly the same compared to the dimer models. The largest difference was around 90 mV, observed between the p(C_6-NDI-T) dimer (-0.54 V vs. SHE) and the trimer models (-0.63 V vs. SHE)."

How were the possible reaction points for O_2 to the polymer backbone chosen? There is no explanation in the text. Why is it not suggested that the NDI may react? In the two NDI-containing polymers, this is where the LUMO will predominately reside; there will be very little wavefunction on the (bi)thiophene, and so it is not clear why O_2 would react on these parts of the systems as drawn. It is also notable that the reactions are only depicted to be on the ends of the oligomers. Are the authors suggesting that O_2 reactions will only take place at chain ends? Given the large number of repeat units (and the O_2 saturation studies), it would be better to represent chemistries that take place in the middle of the representative oligomers.

We thank the reviewer for this very important comment, which prompted us to work on our models further. We computed energies associated with O_2 binding at different sites, including the NDI acceptor unit. The possible binding sites of O_2 with the P-90 dimer model, representing the polymer, were calculated and reported in the new **Figure S8.**

The predicted energy associated with O_2 binding at the center of the dimer unit is 6.9 kcal/mol higher than that for the terminal alkene. The end-on-mode binding of O_2 at the terminal alkene site was predicted to be the most stable, while a monodentate binding of O_2 was found to be far less stable by 22.8 kcal/mol. Binding at the NDI acceptor unit, on the other hand, gives the least stable isomer by 27.1 kcal/mol, suggesting the thiophene unit is the electron carrier to the O_2 substrate. Note that the terminal alkene position (both end-on-mode and monodentate mode) does not represent the real system and only exists because we work with dimer models (we now note this properly in the text to avoid any confusion). Nonetheless, our results conclude that the O_2 binding at the center of the dimer unit is still energetically favorable. We highlight once more that binding to the thiophene unit does not lead to degradation (**Figure S1**), and the conductivity is regained when the film is placed in a deoxygenated environment (**Figure S9a,b**).

We also calculated the interactions between O_2 and the BBL dimer, which has only an acceptor unit in its structure. The predicted free energy of binding to the NDI unit is 2.01 eV (46.5 kcal/mol), thereby ruling out the BBL-O_2 complexation on the NDI unit. We also couldn't locate a stationary point for an O_2-bound complex within the lactone site: the O_2 molecule dissociated despite all our attempts. Experimental observations support these claims: BBL conductivity is not dependent on the presence of O_2 when there are no H^+ ions in the environment (**Figure S9c**).

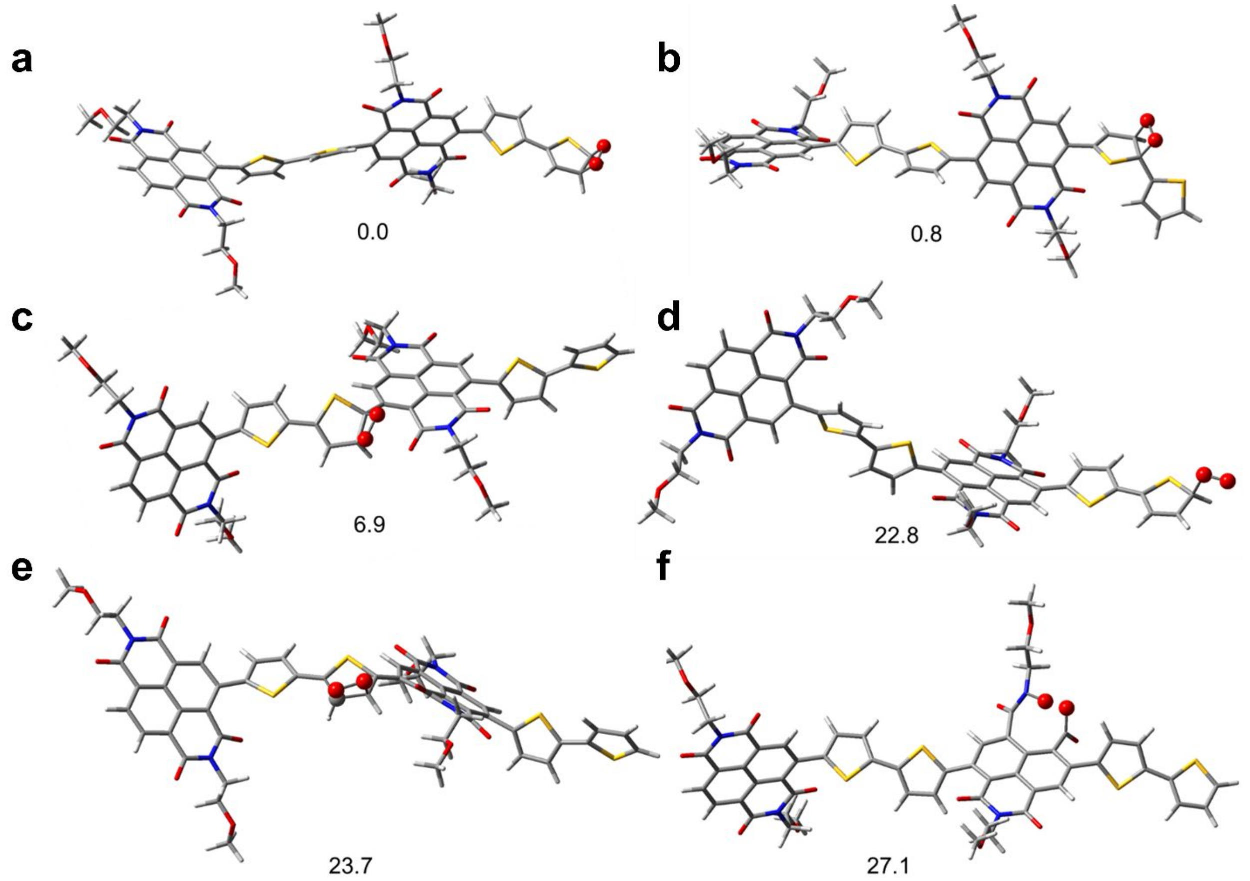

Figure S8. Relative free energies (in kcal/mol) for O_2 binding mode of $POO^{\bullet-}$ species for P-90 dimer.

We modified the manuscript and added the following paragraph in line 288:

"Next, we investigated the binding mode of O_2 to the reduced dimers and the subsequent electron transfer reactions once the film is charged. The reactions are outlined in **equations 1-6**, with the chemical structures of the possible oxygenated species shown in **Figure S8** for P-90. The computed free energy diagram indicates that O_2 has similar binding energies with the reduced dimer models of P-90 and p(C_6-NDI-T) (**Table 1**), likely due to their comparable backbone structures. The predicted energy for O_2 binding to the thiophene adjacent to the end thiophene in the dimer model is 0.8 kcal/mol higher than the energy for O_2 binding to the terminal thiophene in an end-on mode. Meanwhile, the energy for O_2 binding to the thiophene in the middle of the dimer model is slightly higher, i.e., 6.9 kcal/mol more than for the end-on mode. The end-on mode binding of O_2 at the terminal alkene site was predicted to be the most stable, whereas monodentate binding was significantly less stable by 22.8 kcal/mol. Binding to the NDI acceptor unit yielded the least stable isomer, with an energy difference of 27.1 kcal/mol, suggesting that the thiophene unit serves as the electron carrier to the O_2 substrate. It is important to note that the terminal alkene positions (in both end-on and monodentate modes) do not reflect the real system and are artifacts of using dimer models. Therefore, O_2 binding to the thiophene at the center of the dimer unit or adjacent to the terminal thiophene is energetically favorable and most likely the real scenario. Binding at the NDI unit is unfavorable and we hypothesize that it could result in chemical

degradation via ring opening, which would be contradicting with the stable electrochemical performance of P-90 and p(C_6-NDI-T) over multiple cycles (**Figure S1**). The electron transfer upon reduction is thus expected to originate from the polymer's donor unit rather than the NDI."

And in line 337:

"The predicted free energy of binding to the NDI unit is 2.01 eV (46.5 kcal/mol), ruling out the BBL-O_2 complexation on the NDI unit."

Reviewer: 2

Recommendation: Publish in ACS Central Science after minor revisions are noted.

Comments:

The manuscript investigates the issue of oxygen reduction reaction (ORR) affecting the performance and stability of n-type organic mixed ionic-electronic conductors (OMIECs). Specifically, the authors aim to understand how ORR impacts the charge storage performance and operational stability of these materials, which are essential for devices such as bioelectronic sensors, actuators, and soft charge storage systems. Why is this research significant because n-type OMIECs are fundamental to various emerging technologies that interact with aqueous environments. The instability caused by ORR in these materials limits their practical applications, making it imperative to develop strategies to mitigate ORR and enhance device performance and durability. The study focuses on three polymers: P-90, p(C6-NDI-T), and BBL. The authors used the rotating disc electrode (RDE) and linear voltammetry to measure ORR contributions on reduction currents in the polymers. In addition, the authors employed density functional theory (DFT) calculations and X-ray photoelectron spectroscopy (XPS) to analyze the interactions between the polymers and oxygen. Furthermore the authors assessed the performance of organic electrochemical transistors (OECTs) using these polymers under different environmental conditions.

There are some very interesting conclusions.

1. Limited Correlation Between LUMO Levels and ORR: The study found a limited correlation between the lowest unoccupied molecular orbital (LUMO) levels of the polymers and ORR currents, indicating that simply lowering LUMO levels is not sufficient to mitigate ORR.

2. Chemical Moieties' Role: The backbone chemistry of the polymers plays a critical role in controlling O2-related degradation pathways and performance losses.

ORR and Device Functionality: ORR affects the stability and functionality of devices, particularly impacting gate currents without hindering channel currents in OECTs

3. Charge Storage Challenges: Current n-type OMIECs are challenged by fast discharging due to ORR, making them inefficient for charge storage and neuromorphic devices.

Overall , the study is well planned and executed. The conclusion are well supported with experimental data. A deep understanding of the role of oxygen has been achieved. Here are a couple of minor suggestions.

We thank the reviewer for their comments and for summarizing the experiments and findings of the manuscript extremely well. Below, we have addressed the reviewer's comments to the best of our abilities.

To support conclusion 1, the author can have short discussion on n-PBDF, which has a much deeper LUMO than the listed three polymers and also interaction with oxygen and water.

Oxygen dedopes n-PBDF, generating water, while water redopes the polymer and forms hydrogen peroxide. It is a dynamic process.

We thank the reviewer for this interesting suggestion. We acquired PBFDO to explore whether it undergoes ORR. Using a rotating ring disc electrode and the methods mentioned in the "Electrochemical measurements" section of the original manuscript, we evaluated ORR properties of PBFDO. This polymer enables ORR, like the other n-OMIECs tested in the study. ORR currents increase with the increased convection of O_2 towards the film, and the electrode shows lower currents in N_2 saturated environment (**Figure S12**). We determined that PBFDO produces more H_2O than H_2O_2, similar to P-90. We also tested the self-discharge of PBFDO under O_2 and N_2-saturated conditions and found that PBFDO behaves like the other OMIECs tested, self-discharging quickly in the presence of O_2.

Moreover, we conducted similar measurements (**Figure S13**) for P-75, another deep LUMO (4.85 eV) OMIEC. The results show consistent ORR activity and self-discharge behavior, despite the deep LUMO. Note that during the P-75 measurements, we observed that the degradation behavior was both O_2- and voltage-dependent. As a result, we decided to exclude the OECT-based degradation experiments for P-75 from the manuscript and focus solely on the ORR and discharge measurements.

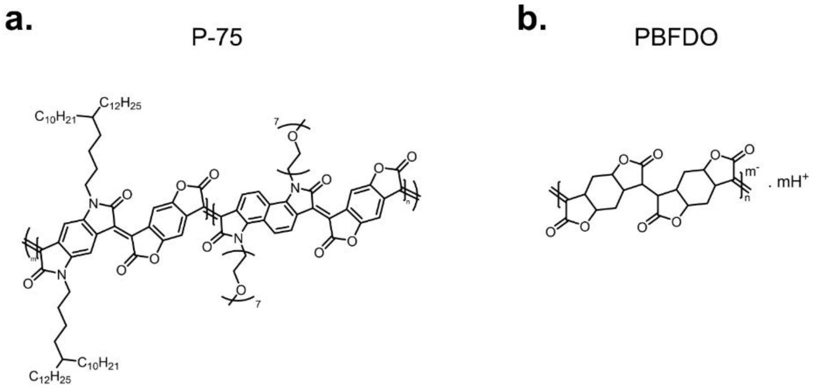

Figure S11. a) The chemical structures of the deep LUMO polymers: P-75 (4.85 eV) and PBFDO (5.18 eV).

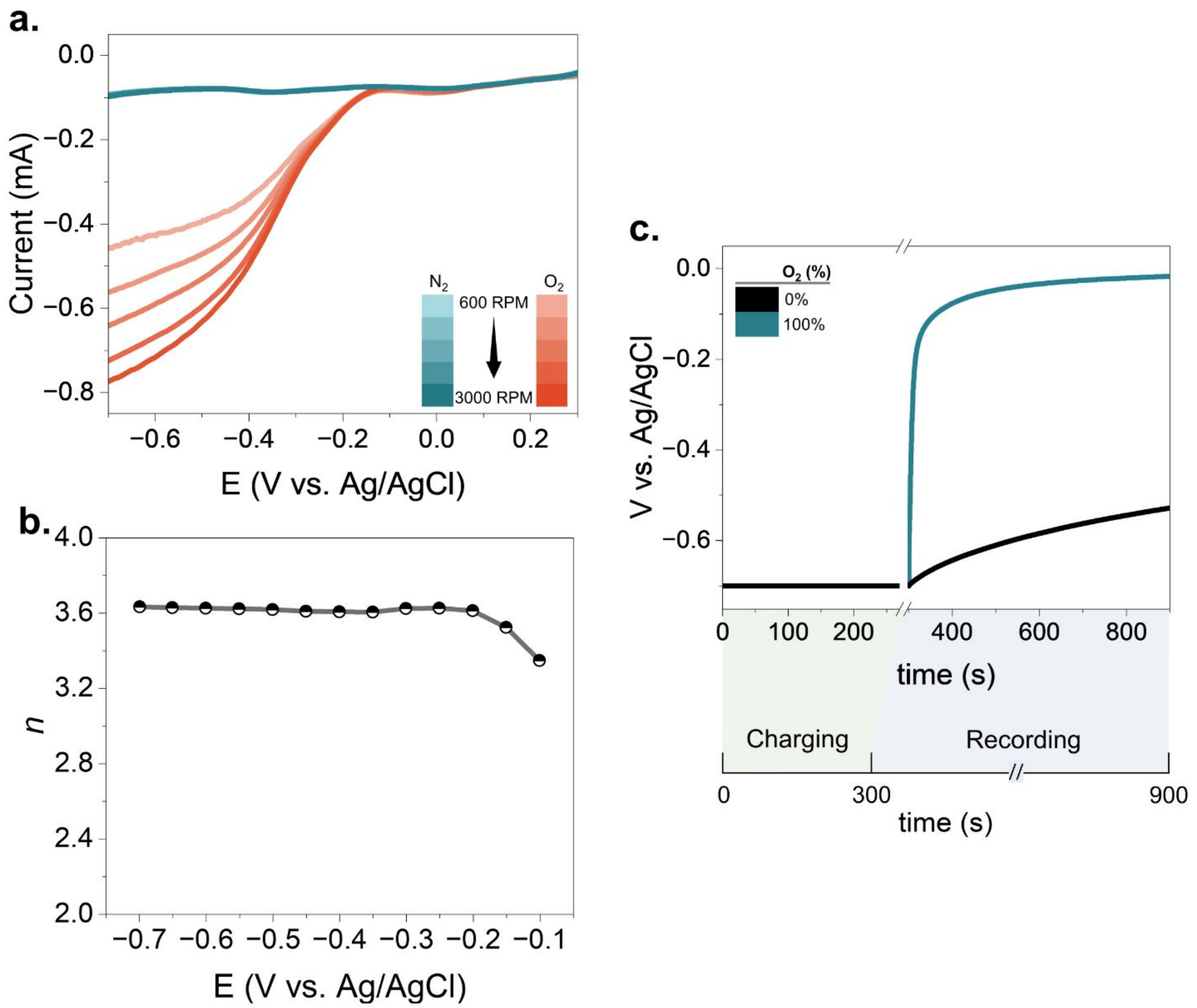

Figure S12. a) Linear sweep voltammograms for PBFDO in N_2- and O_2-saturated PBS at different rotation speeds (600 to 3000 RPM) using a rotating disc electrode. **b)** The number of electrons (n) used to reduce each O_2 molecule versus potential during LSV, recorded for the polymer-coated glassy carbon electrode rotated at 3000 RPM. **c)** The change in the OCP of the PBFDO electrode after being held at -0.7 V vs. Ag/AgCl for 300 seconds in PBS under O_2-saturated and N_2-saturated conditions. All scans were performed at a scan rate of 5 mV/s.

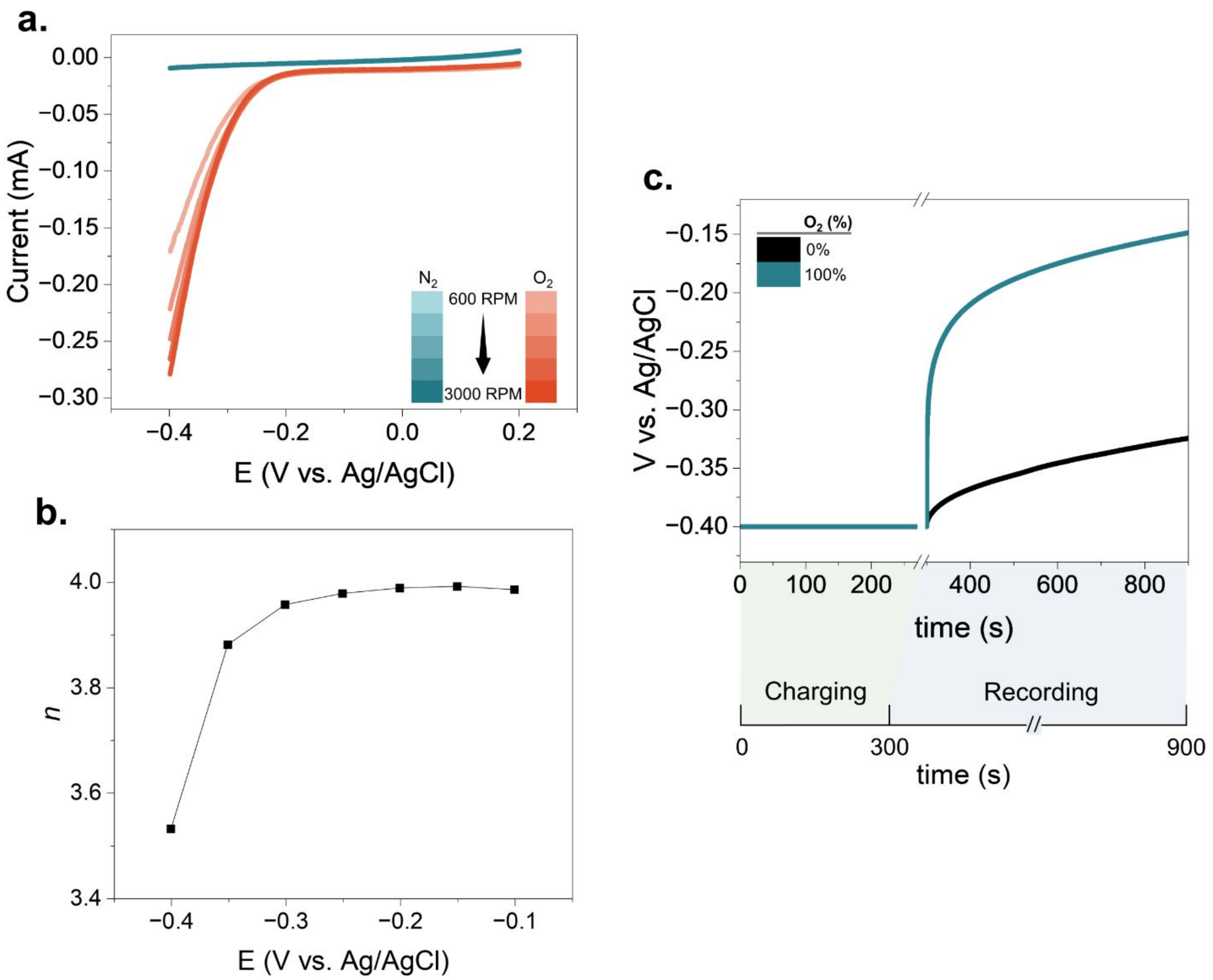

Figure S13. a) Linear sweep voltammograms for P-75 in N_2- and O_2-saturated PBS at different rotation speeds (600 to 3000 RPM) using a rotating disc electrode. **b)** The number of electrons (n) used to reduce each O_2 molecule versus potential during LSV, recorded for the polymer-coated glassy carbon electrodes rotated at 3000 RPM. **c)** The change in the OCP of P-75 electrode after being held at -0.7 V vs. Ag/AgCl for 300 seconds in PBS under O_2-saturated and N_2-saturated conditions. All scans were performed at a scan rate of 5 mV/s.

To describe these results, we add the following discussion in the manuscript in line 351:

"In fact, even polymers with deeper LUMOs, such as p(C2F-V)[40] (4.56 eV), P-75[13] (4.85 eV), and PBFDO[41,42] (5.18 eV) (see chemical structures in **Figure 5a** and **Figure S11**) undergo ORR. As shown in **Figures S12a-b and S13a-b**, both P-75 and PBFDO facilitate ORR, generating mostly water as the reaction byproduct. Self-discharge experiments for all the films reveal their tendency to return to undoped states in air (**Figure 5b**, **Figure S12c and S13c**), similar to shallow LUMO polymers like P-90, p(C_6-NDI-T), and BBL. These results confirm that deeper LUMOs do not necessarily inhibit ORR"

"The next question is whether ORR affects the stability of the reduced films. By recording OECT channel currents for 10 minutes under O_2-free and ambient conditions, we observed that P-90, p(C_6-NDI-T), and BBL-based OECTs exhibited minimal current loss (< 5% after 10 min) in both electrolyte environments (**Figure 5c**). In contrast, p(C2F-V) based OECT experienced a significant decrease in channel currents in the presence of O_2, despite being stable when operated under N_2. Specifically, p(C2F-V) OECT currents decreased by 61.8% in air (**Figure S14**) despite the polymer's deep LUMO."

(40) Wang, Y.; Koklu, A.; Zhong, Y.; Chang, T.; Guo, K.; Zhao, C.; Castillo, T. C. H.; Bu, Z.; Xiao, C.; Yue, W.; Ma, W.; Inal, S. Acceptor Functionalization via Green Chemistry Enables High-Performance N-Type Organic Electrochemical Transistors for Biosensing, Memory Applications. *Adv Funct Mater* **2023**. https://doi.org/10.1002/adfm.202304103.

(41) Tang, H.; Liang, Y.; Liu, C.; Hu, Z.; Deng, Y.; Guo, H.; Yu, Z.; Song, A.; Zhao, H.; Zhao, D.; Zhang, Y.; Guo, X.; Pei, J.; Ma, Y.; Cao, Y.; Huang, F. A Solution-Processed n-Type Conducting Polymer with Ultrahigh Conductivity. *Nature* **2022**, *611* (7935), 271–277. https://doi.org/10.1038/s41586-022-05295-8.

(42) Ke, Z.; Abtahi A.; Hwang J.; Chen K.; Chaudhary J.; Song I.; Perera K.; You L.; Baustert K. N.; Graham K. R.; Mei J. Highly Conductive and Solution-Processable n-Doped Transparent Organic Conductor *J Am Chem Soc* **2023** *145* (6), 3706-3715

For conclusion 3, charging and discharging are intrinsically asymmetrical in conducting polymer, more generally, in redox polymers. Based on the needs, it can be altered. If bistability is truly desired, it can be accomplished through the selection of electrolyte and the electrode modification. Of course, it is difficult to realize in biosensors which have to function in the biological environment.

We agree that charging and discharging are asymmetrical in conducting polymers. However, we believe the reviewer refers to intentional charging and discharging, where a current or voltage is applied to achieve it. In this manuscript, we discuss the "self-discharge" of the electrochemically doped polymer, where a charged film discharges due to its propensity to react with O_2, without any voltage or current applied. Otherwise, the reviewer is correct that the discharge profile can be slower than the charging profile in some materials where ions may be trapped. In our conclusions, we have now emphasized the distinction of discharge within the context of capacitors.

Editorial revisions:

1. **MS File: Associated Content/Supporting Information paragraph is missing.**

This has been added.

2. **MS File: TOC graphic is missing.**

This has been added.

3. **MS File: Synopsis is missing.**

This has been added.

4. **SI File: Pages not numbered as S1, S2, S3, ...**

This has been added.

5. **AU name in MS File: Rajendra Sheelamanthula**

AU name in P+: Sheelamanthula Rajendar

This has been resolved.

oc-2024-00654h.R2

Name: Peer Review Information for "Understanding Oxygen-Induced Reactions and their Impact on n-type Polymeric Mixed Conductor based Devices"

Second Round of Reviewer Comments

Reviewer: 1

Comments to the Author

The authors are to be commended for their careful consideration of the comments and suggestions. The authors sufficiently addressed the comments and the paper should be considered for publication.

Reviewer: 2

Comments to the Author

I am satisfied with the changes made.

Author's Response to Peer Review Comments:

We thank the reviewers for their thorough reviews and suggestions to publish without change after our revision.
